# Survey of mitochondrial sequences integrated into the bovine nuclear genome

**DOI:** 10.1038/s41598-020-59155-4

**Published:** 2020-02-07

**Authors:** Erwin Tramontin Grau, Mathieu Charles, Maureen Féménia, Emmanuelle Rebours, Anne Vaiman, Dominique Rocha

**Affiliations:** 1 Université Paris-Saclay, INRAE, AgroParisTech, GABI, F-78350 Jouy-en-Josas, France; 2 INRAE, SIGENAE, F-78350 Jouy-en-Josas, France

**Keywords:** Mitochondrial genome, Genome evolution

## Abstract

Nuclear copies of the mitochondrial DNA (NUMTs) have already been described in several species. In this context, we identified and analysed 166 bovine NUMT regions with a total length of 430 kbp, representing about 0.02% of the cattle nuclear genome. Copies of all mitochondrial regions were detected in the nuclear genome, with distinct degrees of sequence similarity to the mitogenome. Some NUMT regions include large mitogenome segments and show high similarity to the organelle genome sequence. NUMT regions are frequently modified by insertion of repetitive sequences and by sequence rearrangements. We confirmed the existence of 29 NUMT regions by PCR amplification using DNA from the cow (Dominette) which was used to generate the bovine genome reference sequence, ruling out the possibility that these NUMTs could be artifacts of the genome assembly. As there are NUMT regions with high similarity to the mitogenome, special care is needed when designing primers for mitochondrial DNA amplification. Our results can therefore be used to avoid co-amplification of bovine nuclear sequences similar to mitochondrial DNA.

## Introduction

The endosymbiotic interactions that resulted in the formation of the mitochondria were an important mechanism for eukaryotic cells evolution^1^. During this process, ancestral genes have been transferred from the mitochondrial to the nuclear genome^2^. However, it seems that not all mitochondrial genes have been incorporated into the nuclear genome because of the essential role of this organelle in the bioenergetics functions of the cell^3^.

In this dynamic evolving process, it is not a surprise that NUMTs (nuclear mitochondrial sequences) have been found in the nuclear genome of several species, including cattle, pig, goat and humans^4–9^. It is a continuous DNA transfer process^10^. Some authors consider these NUMTs as molecular fossils that contain important information about the mitogenome evolution^11,12^. Current evidence suggests that interaction between the nuclear and the mitochondrial genomes might be more frequent than expected^13^.

The mode of insertion of the mitochondrial sequences into the nuclear genome is not well understood, but several studies suggest that repair of double-strand breaks is an important mechanism in this process^14,15^. There is evidence that preferential chromosomal positions for mitochondrial insertions are present in the nuclear genome. For example, regions with a higher content of repetitive elements have more chance to be targeted^16,17^. Secondary proliferation in the nucleus is also possible by the duplication of inserted copies^9,18,19^.

The functions of the nuclear mitochondrial copies are still not well-known, but most genes seem to be non-functional (not transcribed), although some could create novel patches of functional exon sequences^20^. Furthermore, depending on the insertion position, NUMTs can have deleterious effects and be associated to diseases^21,22^.

The unintentional analysis of NUMTs as mitogenome sequences has been a potential source of problems of organellar DNA studies^11^. This can generate misleading results in mitochondrial diseases diagnostic, phylogenetic reconstructions, population studies and DNA barcoding analyses^11,23^. Thus, research on the evolution and function of the mitochondrial genome can be compromised if unintended nuclear copies are inadvertently taken into account.

NUMTs discovery has been traditionally performed using BLASTN^24^ with default parameters^4,5,7,8^. There are concerns about the use of this scoring scheme tuned for the alignment between high similarity sequences^16^. However, specific research evaluating the impact of scoring scheme optimization on NUMT discovery is still lacking. In 2012, Tsuji *et al*.^16^ successfully used the program LAST^25^ with an optimized scoring scheme to search for NUMTs, and this methodology has been the choice for some subsequent NUMT studies.

In cattle, the mitochondrial genome has about 16,340 bp and has been the subject of several studies^26^. However, the only comprehensive research on NUMTs was carried out by Liu and Zhao^5^, based on a *Bos taurus* genome reference sequence released in August 2006. A total of 303 NUMTs were identified, with a total length of about 75 kbp. A study with a more recent and improved assembly of the *Bos taurus* nuclear genome could give further information on the mitochondrial sequences present in the nuclear genome.

In this work, we identified and characterized the regions with mitochondrial-like sequences in the nuclear genome using LAST and two assemblies of the bovine genome (ARS-UCD1.2 and UMD3.1.1 versions). Additionally, we tested different methodologies to evaluated the effect of mitochondrial genome linearization and the effect of BLAST scoring scheme optimization on NUMT’s discovery. The information on the identified NUMT regions will enable a more accurate analysis of the cattle mitogenome.

## Materials and Methods

### DNA sequences

Unmasked *Bos taurus* reference genome sequences from the UMD_3.1.1 and ARS-UCD1.2 assemblies were retrieved from Genbank (Assemblies accessions: GCA_000003055.5, 25^th^ November 2014, and GCA_002263795.2, 11^th^ April 2018). As mitochondrial reference sequence, CM008198.1 (Genbank INSDC) was used for all analyses. These sequences were obtained by sequencing the whole genome of L1 Dominette 01449, a Hereford cow^27^.

### Genome-wide search for NUMTs using LAST

Since mitochondrial DNA is circular, it was linearized for alignments. To study the effect of linearization on NUMTs search and to avoid losing NUMTs at the beginning or at the end of the linearized mitochondrial genome sequence, three alternative linearization configurations were tested: (1) a standard linearization starting at position 1 and ending at position 16,340, (2) a standard linearization duplicated in tandem, and (3) a shifted linearization starting arbitrarily at position 8,340 and ending at position 8,339.

These three linearized mitogenome configurations were aligned to the ARS-UCD1.2 and UMD_3.1.1 genome assemblies using version 980 of the program LAST^25^. Following Tsuji *et al*.^16^, the scoring scheme for the alignments was set as: +1 for matches, −1 for mismatches, 7 for gap opening cost and 1 for gap extension cost.

We therefore empirically tested which *e*-value threshold would fit our dataset for the identification of a high number of NUMTs, while being conservative to minimize acceptance of misleading false positives. At first, a set of 1,000 random sequences of 16,340 nucleotides was aligned to both assemblies using LAST with the same NUMT search used above. These random sequences, with specific nucleotide content, were generated using the software Unipro UGENE v1.26.1^28^, based on windows of 1,000 bp of the cattle mitochondrial genome. As a second analysis, *e*-value thresholds of 10^−2^, 10^−3^, 10^−4^ and 10^−5^ were set to filter alignment matches, and thus the impact of different *e*-values thresholds on analyses of our data could be evaluated.

All matches that had an *e*-value below the finally defined threshold and that were placed on chromosomes were considered NUMTs and were analysed further. Matches with an *e*-value above the threshold and/or located in unplaced scaffolds were discarded.

A chi square test was used to verify if the number of alignment matches and the length of the alignment matches per chromosome were proportional to the length of the chromosome. Expected values for each chromosome were calculated by dividing the total number of alignment matches and the total length of the alignment matches by the total length of the genome and multiplying by the length of the respective chromosome.

### Identifying and delimiting NUMT regions

Nuclear copies of the mitogenome might be highly modified by insertions and deletions. Sequences resulting from one NUMT insertion event might be discovered as several alignment matches. Therefore, we merged co-linear NUMTs (matches with *e*-value below the threshold) representing one mitochondrial DNA insertion event. These merged NUMTs were defined as NUMT regions and were named as XXX_YY.ZZ (XXX: for assembly identification; YY: for chromosome; ZZ: for a sequential number). Position of all NUMTs (matches with *e*-value below threshold) were used in this analysis, including matches from alignments with the three mitogenome linearization configurations.

As a first step towards merging NUMTs to NUMT regions, NUMTs that were no more apart than 10 kbp have been grouped as one block of NUMTs. To decide whether each of these nuclear regions encompassed one or several NUMT regions, sequences were visually evaluated for insertions, deletions and inversions using dot plot graphs generated using Unipro UGENE v1.26.1^28^ (x axis: mtDNA; and y axis: nuclear sequence, including additional 1 kbp up- and 1 kbp downstream). Thus, we define a NUMT region as a stretch of nuclear genomic sequence arising from a single mitochondrial DNA insertion event with subsequent modifications by substitutions, insertions, deletions and inversions.

Comparative analysis was performed between NUMT regions identified from alignments to the ARS-UCD1.2 and UMD_3.1.1 genome assemblies. The cattle UMD_3.1.1 genome assembly was first released in 2004, whereas the ARS-UCD1.2 genome assembly was made available in 2018. Therefore, the UMD_3.1.1 genome assembly was the reference genome for cattle genomics studies for almost 15 years. Therefore, comparative analysis of NUMTs search for both assemblies would be valuable to the scientific community. Additionally, concordant results from both assemblies would reinforce the adequateness of the used methodology.

### Comparative analysis between BLAST and LAST for NUMTs discovery

The standard linearization of the mitogenome was aligned to the ARS-UCD1.2 genome assembly using BLAST, in order to perform a comparative analysis with the results obtained using LAST. Two programs were used: BLASTN^24^, version 2.9.0+, optimized for somewhat similar sequences; and, Discontiguous MegaBLAST^24^, version 2.9.0+, optimized for more dissimilar sequences. Default and modified scoring schemes were used, as follows (match/mismatch/gap opening cost/gap extension cost): 2/–3/5/2, 1/–1/4/1, 1/–1/2/1, 1/–1/0/2 for BLASTN; 2/–3/5/2 and 1/–1/4/1 for discontiguous MegaBLAST. The scoring scheme with match/mismatch scores of 2, −3 is tuned for alignments with match identity in the range of 90% and 1, −1 is tuned for alignments with match identity in the range of 75%^29^. Gap costs were set considering that reduction of penalty values increases the likelihood of identification of gapped matches, which are common for older NUMTs. Analyses were performed on the NCBI BLAST platform^30^ using the option for masking regions with low compositional complexity. An *e*-value threshold of 10^−4^ was applied to these BLAST results, and the remaining alignment matches that were no more apart than 10 kbp were merged into one NUMT region. These NUMT regions identified using BLAST were compared to the results obtained using LAST.

### Characterization of NUMT regions

Mitochondrial regions present in each NUMT region were defined based on the positions of the alignment matches in the mitogenome. Information on SNPs located in NUMT regions and in the mitogenome was retrieved from Ensembl^31^, release 96, based on the ARS_UCD1.2 genome assembly. Repetitive regions located in the mitogenome and in the NUMT regions and their flanking positions, 1 kbp up- and downstream, were retrieved from BovineMine, version 1.6^32^, based on the position of the repetitive regions in the ARS_UCD1.2 genome assembly. Open Reading Frames (ORFs) were predicted for all NUMT regions using the program ORF Finder from an NCBI website^33^. For ORFs larger than 300 amino acids and located outside of gene regions, a BLAST alignment with Expressed Sequenced Tags (ESTs) (EST Divisions from Database of GenBank + EMBL + DDBJ sequences) and protein databases (All non-redundant GenBank CDS translations + PDB + SwissProt + PIR + PRF, excluding environmental samples from WGS projects) of *Bos taurus* was performed to verify whether there was evidence that these regions were expressed. In addition, sequences of the 53 NUMT regions overlapping with genes (Ensembl, release 96) were blasted against *Bos taurus* ESTs. All BLAST analyses were performed using the online NCBI BLAST^30^. Potential NUMT duplications were evaluated by inspection of alignments between NUMT regions using Clustal X, version 2.1^34^.

### Experimental validation of NUMT regions

Genomic DNA from the cow (L1 Dominette 01449) which has been used to establish the bovine reference genome assemblies (UMD3.1 and ARS-UCD1.2) was used for the validation of 29 NUMT regions (Supplementary Table S1). A total of 12 of these NUMT regions were also tested and validated for an additional 16 samples, representing seven *Bos taurus* breeds: Aubrac (2), Blonde d’’Aquitaine (3), Holstein (3), Limousin (1), Montbéliarde (3), Normande (2) and Salers (2). Genomic DNA form Dominette was provided by the USDA, ARS, US Meat Animal Center, USA. The genomic DNA from the other 16 samples was sourced by the INRA (Institut National de la Recherche Agronomique).

PCR primers were designed preferentially within the flanking regions of the NUMT regions using Primer-BLAST^35^ and were purchased from Integrated DNA Technologies. Primer sequences can be found in Supplementary Table S1. Polymerase chain reactions were performed in 10 μl, using 50 ng of genomic DNA, 1 U Go*Taq* DNA polymerase (Promega), 1X PCR buffer, 1.5 mM MgCl_2_, 200 μM of each dNTP and 1.0 μM of each PCR primer. The following touchdown cycling protocol was used: 95 °C for 2 min followed by 13 cycles of 95 °C for 1 min, 1 min of annealing (the annealing temperature was progressively lowered from 68 to 56 °C in steps of 1 °C every cycle) and 72 °C for 2 min. These initial cycles were followed by 30 cycles of 95 °C for 1 min, 55 °C for 1 min and 72 °C for 2 min, and a final extension step at 72 °C for 5 min. For amplicons longer than 3 kbp, polymerase chain reactions were performed in 50 μl, using Go*Taq* Long PCR Master Mix (Promega) following the manufacturer’s protocol. The following cycling protocol was used: 95 °C for 2 min followed by 30 to 40 cycles of 92 °C for 30 sec for denaturation, 63 °C for 15 sec for annealing and 65 °C for 10 to 20 min (adjusted at the basis of at least 1 min per kbp) for extension, and a 65 °C final extension for 20 min was applied. PCR products were analyzed by electrophoresis on an 0.8% agarose gel to verify the expected length of the selected amplicons. The nucleotide sequence of some of the amplicons were subsequently determined using Sanger sequencing (Eurofins Genomics). All sequences were visually inspected using Chromas (Technelysium) and then aligned to the ARS-UCD1.2 cattle reference genome sequence using BLASTN.

## Results

In brief, the workflow to identify NUMT regions comprised three main steps: (1) align the linearized mitogenome to the nuclear genome; (2) define an *e*-value threshold and select alignment matches with *e*-values below this threshold for further analysis (matches with *e*-values below the threshold represent the cattle NUMTs); and, (3) merge co-linear matches (or NUMTs) into joined NUMT regions. Additionally, we performed comparative analyses and characterization of the NUMT regions obtained.

### Genome-wide search for NUMTs using LAST

LAST was prioritized as alignment tool as its scoring scheme can be adjusted to alignment of low similarity sequences, which is expected for old NUMTs^6,16^. This tool was applied in recent studies of NUMTs and it seems to perform better than BLASTN^6,16^.

The *e*-value threshold was set with the aim of minimizing interference from small unspecific or random alignment matches, while maximizing the identification of NUMT regions. At first, 1,000 random sequences were aligned to the UMD3.1.1 and ARS-UCD1.2 genome assemblies using LAST. A total of six alignment matches were detected. The lowest *e*-value was 6.5 × 10^−4^ and the alignment matches length ranged from 49 to 62 nucleotides. Additionally, the impact of setting different *e*-values thresholds was evaluated (Supplementary Table S2). The decrease of the *e*-value threshold, within the range from 10^−2^ to 10^−5^, had little impact on the number of NUMTs detected, as only few alignment matches were excluded. Therefore, an *e*-value threshold of 10^−4^, comparable to other studies of NUMTs^4,5,16^, was applied for all further analyses.

The three linearized configurations of the mitogenome were aligned to the UMD3.1.1 and ARS-UCD1.2 genome assemblies using LAST. Results for both genome assemblies (Table 1) indicate that linearization has little impact on the total alignment of the NUMTs detected in the cattle nuclear genome. For both assemblies, total alignment length was slightly higher (by a few hundred nucleotides) when the duplicated linearization configuration of the mitochondrial genome was used. NUMTs spanning the region of the linearization cut-point were correctly detected for alignments implemented with all three linearization configurations.

**Table 1:** Results obtained using LAST for alignment with both genome assemblies and three different mitogenome sequence linearization configurations.

Assembly	Mitogenome linearization configuration	Alignments^a^					
		Number	Length (kbp)	Mitochondrial (kbp)	Nuclear (kbp)	Gaps (kbp)	Identity (%)
UMD3.1.1	Standard	439	244.9	243.2	239.6	2.1	78.2%
	Duplicated	431	246.7	244.6	241.3	2.1	78.1%
	Shifted	441	246.2	244.5	240.8	2.1	78.2%
ARS-UCD1.2	Standard	426	241.5	239.8	236.3	2.0	77.9%
	Duplicated	419	243.1	241.4	237.9	2.1	77.9%
	Shifted	428	242.8	241.1	237.6	2.1	77.9%

^a^Information about the number of alignments, total alignment length, total length of mitochondrial genome aligned, total length of nuclear genome aligned, alignment gap and average identity between mitochondrial and nuclear sequences. An e-value threshold of 10^−4^ was applied to select data to be included in the calculations. For the duplicated linearization, duplicated alignments were excluded for calculations.

**Figure 1:**
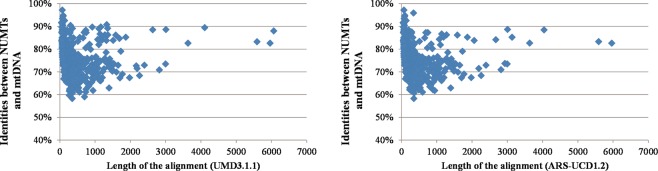
Sequence identities between nuclear and mitochondrial DNA (mtDNA) across the total length of the alignments. Alignments from standard mitogenome linearization against the UMD3.1.1 (left) and against the ARS-UCD1.2 (right) genome assemblies. Footnote: For the ARS-UCD1.2, one alignment match of 11,722 bp is not shown in the graph.

**Figure 2:**
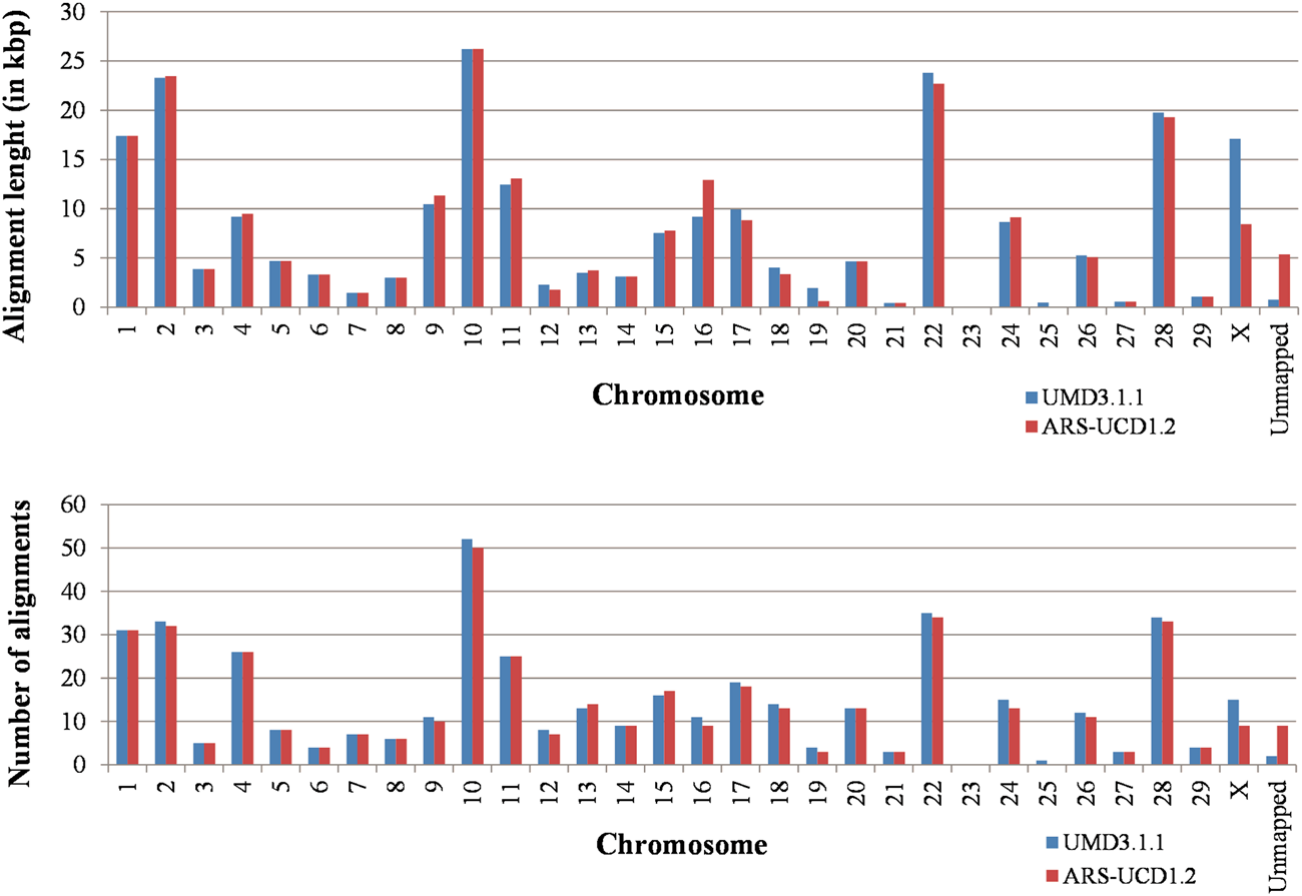
Number of alignments between nuclear and mitochondrial DNA per chromosome (bottom) and total alignment length per chromosome (top) from the alignment with the standard mitogenome linearization using LAST.

Alignment results (Table 1) indicate little difference of NUMTs identified between the UMD3.1.1 and ARS-UCD1.2 genome assemblies. For both, similarity between nuclear and mitochondrial sequences ranged between 58% and 97% (Fig. 1), with an average of about 78% (Table 1). Alignment matches were detected in 28 chromosomes for the UMD3.1.1 genome assembly and in 27 chromosomes for the ARS-UCD1.2 genome assembly (Fig. 2 and Supplementary Table S3). No matches were identified on chromosome 23 for any of the genome assemblies. The similarity between NUMTs and mitogenome sequences from larger alignment matches is usually high, whereas there is more similarity variation for shorter alignment matches (Fig. 1). It is important to note that the number and the total length of the alignment matches were not proportional to the length of each chromosome (*P*-value = 0, chi square test).

For the UMD3.1.1 genome assembly, the total alignment length was 246.7 kbp, with alignment matches ranging from 55 to 6,065 bp. The longest alignment match was obtained for all three mitogenome linearization configurations. For the ARS-UCD1.2 genome assembly, the total alignment length was 243.1 kbp, with alignment matches ranging from 48 to 11,856 bp. The longest alignment match was obtained using the duplicated linearization of the mitochondrial genome (this NUMT was detected in more than one match for the other two linearization configurations). For both assemblies, more than 95% of the alignment matches were shorter than 2 kbp.

### Identifying and delimiting NUMT regions using LAST

**Figure 3:**
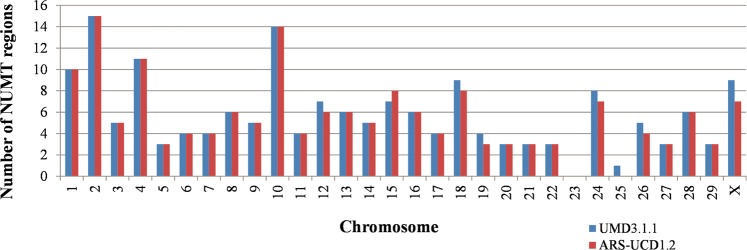
Distribution of the NUMT regions across all chromosomes for both bovine genome assemblies.

Alignments performed using LAST allowed the identification of 173 and 166 NUMT regions, with a total length of 441.8 kbp and 430.3 kbp, for the UMD3.1.1 and ARS-UCD1.2 genome assemblies respectively (Table 2 and Supplementary Table S4). NUMT regions were discovered in 28 chromosomes for the UMD3.1.1 and in 27 chromosomes for the ARS-UCD1.2 genome assembly (Supplementary Table S5 and Fig. 3). NUMT regions do not form clusters. Different linearization configurations had little impact on alignments, most NUMT regions were detected in the three cases. The exceptions, for both assemblies, were for two 55 nucleotides-long NUMT regions, located on chromosomes 1 and 21, that were not detected when the duplicated linearization was used.

**Table 2:** NUMT regions information defined using LAST results, by NUMT region length range.

Length Range^a^	UMD3.1.1 genome assembly^b^				ARS-UCD1.2 genome assembly^b^			
	NUMT Regions	% of Total	Total length (bp)	% of Total	NUMT Regions	% of Total	Total length (bp)	% of Total
55–100	25	14.5%	1,860	0.4%	25	15.1%	1,860	0.4%
101–300	51	29.5%	8,783	1.9%	52	31.3%	9,018	2.1%
301–1000	35	20.2%	17,519	3.7%	34	20.5%	16,351	3.8%
1001–5000	36	20.8%	87,882	19.5%	30	18.1%	77,919	18.1%
>5000	26	15.0%	325,835	74.4%	25	15.1%	325,116	75.6%
Total	173	100.0%	441,789	100.0%	166	100.0%	430,264	100.0%

^a^Length range of the NUMT regions.

^b^Information about the number of NUMT regions per length range, % of the number of NUMT regions per length range, total length of the NUMT regions per length range, and % of the total length of the NUMT region per length range.

For both assemblies, NUMT regions length ranged from 55 bp to about 34,830 bp. NUMT regions with more than 5,000 bp, although representing only around one sixth of the total number of NUMTs regions, comprise around three quarters of the NUMT regions total length (Table 2). On the other hand, NUMT regions smaller than 300 bp comprise only about 2.5% of the total NUMT regions length, but represent almost half of the number of NUMT regions (Table 2). A total of 8 NUMT regions detected on UMD3.1.1 were not present in ARS-UCD1.2, while conversely one NUMT region recorded for the ARS-UCD1.2 could not be detected in UMD3.1.1.

Although the total alignment length reached around 240 kbp for both assemblies, the total length of the NUMT regions reached 430–440 kbp, representing 0.02% of the cattle nuclear genome. For both assemblies, this represents a difference of about 200 kbp between the total length of NUMTs and NUMT regions. An almost twofold increase of the total length detected after merging co-linear NUMTs. It is important to note that NUMT regions ARS_14.2 and ARS_14.3 might represent only one long and highly modified NUMT region, as well as NUMT regions ARS_X.8 and ARS_X.9 (Supplementary Fig. S1).

**Figure 4:**
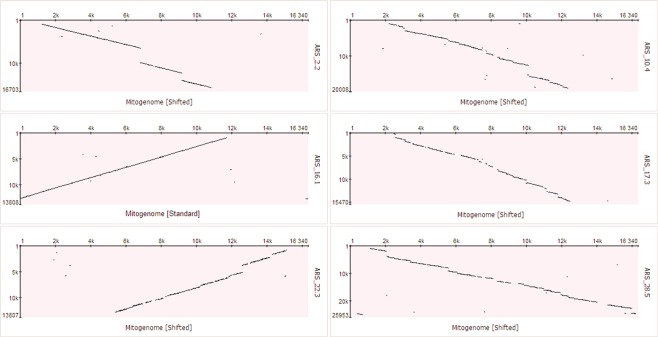
Dot plots representing the alignment of large NUMT regions with the bovine mitogenome, based on results obtained from NUMTs discovered with the ARS_UCD1.2 genome assembly. Mitochondrial DNA sequence is plotted on X axis and NUMT regions are plotted on Y axis. The positions indicated in the axes of the dot plots start at 1 and go to the complete length of the sequence. Therefore, dot plot representations are not in the same scale for the Y axis and the positions of the shifted representation of the mitochondrial DNA is not adjusted for differing linearization cut-points.

**Figure 5:**
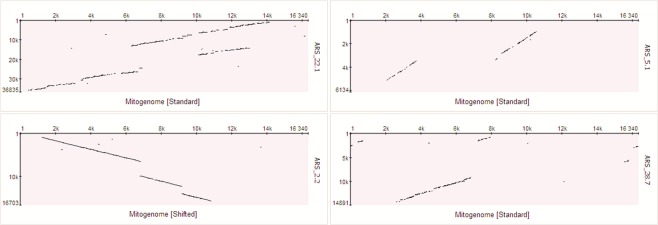
Dot plots of alignments between nuclear and mitochondrial DNA representing partial duplications (upper left, ARS_22.1), deletions (upper right, ARS_5.1) and insertions (bottom, ARS_2.2 and ARS_28.7). Examples are based on results obtained from NUMTs discovered with the ARS_UCD1.2genome assembly. Mitochondrial DNA sequence is plotted on X axis and NUMT regions are plotted on Y axis. The positions indicated in the axes of the dot plots start at 1 and go to the complete length of the sequence. Therefore, dot plot representations are not in the same scale for the Y axis and the positions of the shifted representation of the mitochondrial DNA is not adjusted for differing linearization cut-points.

About 8% of the alignment matches between mitochondrial and nuclear sequences show sequence similarity higher than 85%. For example, NUMT region ARS_16.1, with a total length of 11,809 bp, was detected in one contiguous alignment match, including only 19 gapped positions and showing similarity of 88.8% to the mitochondrial sequence. It spans, without rearrangements, from position 16,207 to position 11,706 of the mitogenome (Fig. 4, middle-left). Further examples of large NUMT regions with higher similarity to the mitogenome sequence are shown in Fig. 4. NUMT regions showing sequence rearrangements, mostly insertions and deletions, are shown in Fig. 5.

We analysed two genome assemblies to verify that the results were comparable. In the following analyses, only NUMT regions from the ARS-UCD1.2 genome assembly will be characterized.

### Genome-wide search, identification and delimitation of NUMT regions using BLAST

NUMTs with different degrees of divergence from the mitogenome sequence are detected in the nucleus, from highly conserved to highly modified copies. Previously, BLASTN with default parameters was used in several NUMTs studies^5^. As this default scoring scheme is tuned for alignments of sequences with high similarity, there are concerns that a relevant number of divergent NUMTs might be not identified^16^. In this context, we tested different BLAST scoring schemes suitable for alignments of sequences with different degrees of divergence. BLAST alignments were performed using standard linearization of the mitogenome and the ARS-UCD1.2 genome assembly.

Alignment programs, scoring scheme and results are summarized in Table 3. Total length of alignment matches ranged from 211.1 kbp to 316.2 kbp, depending on the scoring scheme of the analyses. A total of 208 NUMT regions, with a total length of 510.5 kbp, were detected when the alignment results obtained with all BLAST strategies were considered together. When each search strategy was considered individually, the total length of NUMT regions ranged from 356.7 kbp to 495.4 kbp. As expected, when gap costs are lowered, smaller NUMTs showing low similarity to the mitogenome sequence were identified, some of these could not be validated by dot plot visual inspection.

**Table 3:** Alignment results from different BLAST strategies using the ARS-UCD1.2 genome assembly and standard linearization configuration of the mitochondrial DNA.

BLAST			Sequence linearization	Alignments^d^			NUMT Regions^e^	
#^a^	Program	Scoring^c^		Number	Length (kbp)	Identity (%)	Number	Length (kbp)
1	Discontiguous MegaBLAST	2/–3/5/2	Standard	283	211.0	79%	115	356.7
2	Discontiguous MegaBLAST	1/–1/4/1	Standard	289	219.7	77%	121	368.1
3^b^	BLASTN	2/–3/5/2	Standard	431	252.4	78%	170	436.0
4	BLASTN	1/–1/4/1	Standard	478	290.2	75%	190	478.6
5	BLASTN	1/–1/2/1	Standard	471	311.5	75%	192	495.3
6	BLASTN	1/–1/0/2	Standard	460	316.2	76%	190	495.4
Consolidated results of all BLAST searches							208	510.5

^a^BLAST # is the identification of the BLAST strategy used.

^b^BLAST # 3 is called as “default BLAST” from here on.

^c^Scoring scheme: match/mismatch/gap opening cost/gap extension cost.

^d^Information about number of alignments, total alignment length and identity between sequences.

^e^Information about number of NUMT regions and total their total length. NUMT regions located on unplaced scaffolds were discarded from these analyses.

Comparing LAST and BLAST results, the total length of the NUMT regions of 430.3 kbp detected using LAST is similar to the 436.0 kbp NUMT region detected using BLASTN with the default scoring. When results of all BLAST strategies are included, the difference in the length would be higher, since a total length of 510.5 kbp was recorded for NUMT regions detected. NUMT regions exclusively detected by LAST or by BLAST searches are listed in Supplementary Tables S6 and S7. Most of these regions are shorter than 1 kbp.

### NUMT regions characterization

**Figure 6:**
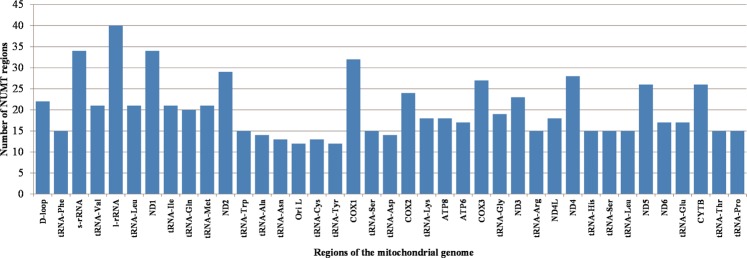
Number of NUMT regions containing, completely or partially, each region of the bovine mitogenome.

All mitochondrial genes had alignment matches with NUMT regions, but distinct numbers of copies were detected (Fig. 6). On average, we found that each mitochondrial region had similarities to 20 different NUMT regions. *COX1* and *ND1* occur in higher frequency, as they were detected 34 and 32 times. *CYTB* and D-loop region had homology to 26 and 22 NUMT regions, respectively. Nuclear copies highly similar to the mitochondrial sequence were detected for several NUMT regions. For example, NUMT region ARS_16.1 was discovered with high sequence similarity to the mitogenome in one contiguous alignment match, which includes several mitochondrial genes (*e*.*g*., D-Loop; *ND 1*, *ND2*, *ND3*, *ND4l* and *ND4*; *ATP6* and *ATP8*; and, *COX1*, *COX2* and *COX3*). There are some NUMT regions that also comprise several mitochondrial genes.

In the mitochondrial genome, only four regions of repetitive sequences were detected, comprising 255 bp. This contrasts with the high number of repetitive sequences identified in NUMT regions: 681 repetitive sequences, comprising 160.8 kbp, were found. In this context, one can verify that the differences detected between the total length of the NUMT regions (430.3 kbp, representing the merged matches) and the total length of the NUMTs (about 247 kbp, representing the alignment matches between nuclear and mitochondrial sequences) is due to insertion of repetitive sequences within the NUMT regions. SINEs (Short interspersed nuclear elements) and LINEs (Long interspersed nuclear elements) are the most frequent classes of repetitive elements detected within NUMT regions, distributed within 522 repetitive sequences and covering a total length of 148.4 kbp. There is also a significant number of LTRs (Long terminal repeats), comprising a total length of 6.2 kbp. Detailed information about repetitive sequences in cattle NUMT regions is shown in Supplementary Table S8.

Regarding the insertion position of NUMT regions, an average of about 48% of their flanking sequences (considering 1 kbp upstream and 1 kbp downstream) contain repetitive elements. We could identify at least 11 NUMT regions that present more than 75% of repetitive sequences on both of their flanking regions. On the other hand, at least 20 NUMT regions showed less than 25% of repetitive sequences sites up- and downstream. DNA transposons and LTRs are more frequent in flanking regions, whereas SINEs and LINEs are more frequent within NUMT regions. Furthermore, 53 NUMT regions were found within boundaries of 58 genes (Supplementary Table S9).

For the whole mitochondrial genome, 1,163 SNPs (single nucleotide polymorphisms) have been annotated (Ensembl release 96), about one SNP per 14 nucleotides. As mitochondria have a small genome that is easier to sequence and explore than the nuclear genome, it could be expected that a significant portion of the organellar SNPs have already been identified. On the other hand, about 19,000 SNPs were found in NUMT regions. This is a high number, as until now no specific study has been performed to identify polymorphic sites in these genomic regions. For the whole cattle nuclear genome, about 97 million SNPs were detected (Ensembl release 96), corresponding to approximately one SNP per 27 nucleotides, considering the total length of the 2.63 Gbp of the sequences assigned to a chromosome in the ARS-UCD1.2 genome assembly. SNPs were detected with a significant higher frequency in NUMT regions (about one SNP per 23 nucleotides) (*p*-value < 0.00001, Fisher Exact test).

### Nuclear duplications as secondary insertion events

Alignments between NUMT regions, including their flanking sequences, allowed the identification of potential duplication events of these nuclear copies. Some NUMT regions, which presented high similarity sequences between each other and in their flanking regions, seem to have undergone one or more events of duplication during their evolution in the nucleus, as example of ARS_10.7 and ARS_10.8 (Supplementary Fig. S2), and ARS_18.6, ARS_18.7 and ARS_18.8 (Supplementary Fig. S3). Furthermore, we identified similar repetitive sequences flanking this potentially duplicated regions, which reinforces the possibility of nuclear duplication after the nuclear insertion (Supplementary Figs. S2 and S3).

### *In silico* analysis of the expression of nuclear copies of mitochondrial genes

To investigate the possible expression of proteins encoded by the mitochondrial nuclear sequences, a total of 3,669 ORFs, ranging from 25 to 434 amino acids, were predicted within the detected NUMT regions.

Focusing on ORFs larger than 300 amino acids that did not overlap with protein-coding genes, two ORFs originating from ARS_12.1 and comprising a total of 773 amino acids were detected. They displayed 97% of similarity to an endonuclease reverse transcriptase (AAY53484.1) of 1,272 amino acids and also to some other mRNA sequences (*e*.*g*., EH202937.1). An alignment between the endonuclease sequence AAY53484.1 and the mRNA EH202937.1 resulted in similarity >99%. This indicates that the portion of the endonuclease sequence within the NUMT region is probably not expressed, as it has lower similarity to this mRNA EH202937.1 (97%).

To search for possible transcripts, the 53 NUMT regions overlapping with 58 genes (127 transcripts) were blasted against *Bos taurus* ESTs. Part of the NUMT region ARS_22.3 aligned with >99,5% of similarity to three ESTs (CB460908.1, CB459118.1 and BM031821.1), two of them overlapping each other. NUMT region ARS_22_3 is located within intron 4–5 of the gene encoding choline dehydrogenase (transcript ENSBTAT00000013227.5). These three ESTs did not show high similarity to the mitochondrial sequence, which allows ruling out that they originated from mitochondrial genome transcription. Furthermore, these three ESTs aligned with high similarity to only one position of the nuclear cattle genome. Thus, these findings indicate the possibility that some NUMT regions might be expressed, but further investigation of this result is needed, as only a small number of ESTs were detected and contamination of the original cDNA library with genomic (nuclear) DNA cannot be ruled out. Expression can only be experimentally confirmed.

### Experimental validation of NUMT regions

We randomly selected 29 NUMT regions from 18 different chromosomes for experimental validation. All 29 amplicons were of expected length when using DNA from Dominette (Supplementary Table S1), indicating no genome assembly artifacts (Supplementary Fig. S4). The smallest twelve NUMT regions out of these 29 were analysed further with 16 additional DNA samples, representing seven *Bos taurus* breeds. The amplicons were of the expected length (Supplementary Fig. S5). The sequence of four of these amplicons (NUMT regions UMD_2.5, UMD_10.8, UMD_15.7 and UMD_15.B) were also checked by sequencing with DNA from animals representing five different breeds (Aubrac, Blonde d’Aquitaine, Holstein, Montbéliarde and Normande). Sequence alignments confirmed the presence of these NUMT regions. In addition, the lack of genetic variability in these amplicons among the different animals tested suggest that the insertion of these nuclear mitochondrial sequences is older than the differentiation between these breeds. An example is showed in Supplementary Fig. S6.

## Discussion

Mitochondrial DNA sequences are widely used in population and phylogenetic research^11,26,36^. They are also basis for other genetics studies, including heteroplasmy^11^. Several mitochondrial genes, such as *COX1*, *CYTB*, *12S rRNA* and *16S rRNA*, are frequently used in barcoding analyses^37^. Mitochondrial-like sequences in the nuclear genome can influence the results of these molecular genetics studies. As the identification of NUMTs has grown with the sequencing of new genomes^11^, the analyses implemented here are aimed at identifying and characterizing NUMTs in cattle, to provide information for further studies on NUMTs and mitochondrial DNA.

Our comparative study indicates that, in general, minor differences on NUMTs identification are recorded when using different linearization configurations of the mitogenome and the UMD_3.1.1 (from 2014) or ARS-UCD1.2 (from 2018) genome assemblies. The number and the total length of the NUMT regions are slightly higher with the UMD3.1.1 (173 regions; 441.9 kbp) compared to the ARS-UCD1.2 genome assembly (166 regions; 430.3 kbp). A total of 94% of the regions were identified in both assemblies. This indicates that the updates of the new assembly did not much change the identification of NUMTs in the bovine genome and suggest that these mitochondrial-like sequences in the nuclear genome are not genome assembly artifacts.

Although our NUMTs discovery was implemented using LAST, we performed comparative analysis on NUMTs search using BLAST. For several previous studies on NUMTs, BLASTN with default parameters was used^5,7–9,38^. We tested changes to the scoring scheme of BLAST searches. This allowed to increase the detection of NUMTs up to a total length of 510.5 kbp, comprising 208 NUMT regions, including 56 NUMT regions that were not detected with LAST. However, some highly divergent NUMTs of short length were discovered, some of which could not be validated by dot plot visual analysis. For our data, BLASTN with scoring scheme 1/–1/4/1 seems to be a suitable alternative option to LAST, as it allows the detection of a large number of NUMT regions. In conclusion, both programs, LAST and BLAST, seem to be appropriate for identification of NUMTs. Further studies on optimization of the scoring scheme could improve the detection of NUMTs with distinct degrees of sequence similarity to the mitogenome.

In 2007, Liu and Zhao^5^ published a comprehensive study of cattle NUMTs. NUMTs discovery was implemented using BLASTN with default parameters and a partial unassembled version of the nuclear *Bos taurus* genome. A total of 355 NUMTs (alignment matches) were identified, which were merged into 303 NUMT regions, ranging from 37 to 1932 bp and comprising a total length of 75.4 kbp. Two additional studies on NUMTs included cattle in their sampling, both used BLASTN and UMD3.1.1 genome assembly. Hazkani-Covo *et al*.^11^, sampling 85 species, identified 279 NUMTs (alignment matches) in cattle, with a total length of 70 kbp. Calabrese *et al*.^9^, studying NUMTs colonization in mammalian genomes, identified 432 NUMT regions (considering merged NUMTs) in cattle, comprising slightly less than 0.01% of the genome. We detected about 240 kbp of NUMTs (alignment matches) and 173 NUMT regions (merged NUMTs) comprising 441.9 kbp for the UMD3.1.1 genome assembly. Therefore, in our study, NUMT regions represent about 0.02% of the cattle genome. Our results, as those from Calabrese *et al*., represent an increase on the total length of detected NUMTs. The lower number of NUMT regions in our study might indicate that our NUMTs merging procedure, with visual inspection using dot plots, was more effective to identify single insertion events.

NUMT regions are present in 28 cattle chromosomes and did not form clusters. This is in accordance with Tsuji *et al*.^16^, who studied NUMTs in mammals and did not identify clusters. NUMT regions with rearrangements were detected, including duplications, insertions and deletions. These rearrangements are expected, as most NUMT regions tend to be non-functional sequences that could undergo genetic drift. Tourmen *et al*.^8^, studying the human genome, found a variety of rearrangements and estimated that 7% of the NUMT regions contained inversion events or sequence displacements.

NUMT regions detected using LAST alignment on the ARS-UCD1.2 genome assembly were further analysed. All mitochondrial regions were detected in the NUMT regions, with distinct number of copies. Some regions that are frequently used in phylogenetic and population studies^36,39^, such as D-loop and *CYTB*, which are present in several NUMT regions. Therefore, special attention should be given for the selection of primers for PCR amplification of these mitochondrial genes. Since nuclear and mitochondrial copies have different evolutionary histories, including distinct mutation rates and selection pressures, results could be obtained that could affect important resources and duration of scientific research.

Some studies discussed the under-representation of the D-loop nuclear copies, such as in human^16^. In the mitogenome, the D-loop tends to present higher mutation rate than other mitochondrial regions^40^. This could result in higher divergence between nuclear and mitochondrial sequences of this region. Consequently, lower number of D-loop NUMTs would be identified. Alternatively, the lower occurrence of D-loop NUMTs could result from preferential nuclear insertion of other mitochondrial DNA regions. In our study, the D-loop is well represented, as much as other sections of the organellar DNA.

Considerable numbers of repetitive elements sequences are present in NUMT regions (681 covering 160.8 kbp). It can be shown that most of the difference detected between the total length of the NUMT regions (430.3 kbp) and the total length of the NUMTs (about 243 kbp) might be due to the insertion of repetitive sequences within the NUMT regions. Dot plot graph analysis helps to understand this difference, as they indicate several insertions within NUMT regions. This suggests that the evolution of NUMT regions is highly influenced by the insertion of repetitive sequence classes. SINEs and LINEs are the most frequent repetitive sequences classes. This is in agreement with findings of Tourmen *et al*.^8^, who also identified small intercalated repetitive sequences (mostly SINEs and LINEs) within human NUMT regions.

The frequency of SNPs in NUMT regions is significantly higher than the average found in the nuclear genome. On average, about one SNP was detected per 23 nucleotides of the NUMT regions compared to 27 for the nuclear genome. This suggests that these regions have a higher mutation rate than other regions of the genome. An important contributing source of substitutions in NUMTs could be that they probably for the most part are non-functional and can change in an unconstrained fashion. Alternatively, the higher frequency of SNPs in NUMT regions compared to the other genome regions might be due to a high error rate in calling polymorphisms in the NUMT regions, because of misalignments with other NUMT sequences or true mitochondrial sequences. This should be further studied, as it might have impact on nuclear and mitochondrial SNPs studies.

On average, 48% of the sites flanking NUMT regions (considering 1 kbp upstream and 1 kbp downstream) contain repetitive sequences. As the total masked regions of the cattle genome comprise 49.38%, including simple/tandem repetitive sequences, satellite DNA, and low complexity regions, we cannot conclude that NUMTs are preferentially inserted in regions with a high percentage of repetitive sequences and, thus a more detailed study should be done. This result is in contrast to some other studies, such as in human, which indicated a tendency toward the presence of NUMTs in regions with higher content of repetitive sequences^16^. We could identify at least 11 NUMT regions with higher content of repetitive sequences (≥75%) on their two flanking regions. On the other hand, at least 20 NUMT regions display a lower repeat content (≤25%) in their upstream and downstream sites. Schiavo *et al*.^6^ also identified NUMTs in pig that were not inserted in regions with repetitive sequences.

We identified NUMT regions that might affect the function of genes, as 53 of them overlap with genes. For example, ARS_15.6, which is 5,296 bp-long is located completely within intron 45–46 (5,876 bp-long) of the myosin VIIA gene (*MYO7A*, ENSBTAT00000071696.1). Further studies should be done, as this type of interference has already been shown to be correlated to deleterious effects and associated with diseases^21,22^.

Cases of subsequent duplication of NUMTs in the nuclear genome were identified in other species, such as in cats^41^ and humans^8^, and it seems that many bovine NUMT regions are originated from this process^16^. This might be a relevant mechanism for colonization of the nuclear genome by NUMTs. We detected some NUMT regions that might be originated by nuclear sequence duplication, but it is not possible to conclude that this would be a recurrent mechanism of the multiplication of NUMTs in the cattle genome.

Our results are supported by the experimental validation of 29 NUMT regions with DNA from the cow, which the two bovine genome reference assemblies were derived from. In addition, 12 of these NUMT regions were experimentally validated with DNA samples from seven other *Bos taurus* breeds. Consequently, it can be concluded that that these NUMT regions are not assembly artifacts and that the insertion of these mitochondrial sequences into the nuclear genome is older than the differentiation of these breeds.

In summary, we detected NUMTs presenting a wide range of lengths and different degrees of similarity to the mitogenome sequence. Comparable results were obtained using three linearization configurations of the mitogenome and two distinct cattle assemblies. Furthermore, similar results were obtained when using LAST or BLAST programs. These results suggest that we used an adequate strategy for NUMT’s identification.

Interestingly, we found several NUMT regions showing high similarity to the mitochondrial DNA (some of them comprising almost the complete mitochondrial genome) that potentially could pose a risk to mitochondrial DNA studies^11,23^. Furthermore, some NUMT regions might impact the analysis of mitochondrial and nuclear SNPs, and this should be investigated in further studies.

## Supplementary information

Supplementary Information.
