# Supplementary material for: Survey of mitochondrial sequences integrated into the bovine nuclear genome
Source: Sci Rep. 2020 Feb 7;10:2077. doi: 10.1038/s41598-020-59155-4 (PMC7005759; doi:10.1038/s41598-020-59155-4)
Supplement: Supplementary file 1 — Supplementary Information. [file 41598_2020_59155_MOESM1_ESM.pdf]

Survey of mitochondrial sequences integrated into the bovine nuclear genome

Grau, E.T., Charles, M., Féménia, M., Rebours, E., Vaiman, A. and Rocha, D.

Supplementary information

Table S1. NUMT regions selected for validation.

Table S2. Estimation of the impact of *e*-value thresholds for alignments between nuclear assembly (UMD3.1.1 and ARS-UCD1.2) and standard mitogenome linearization using LAST.

Table S3. Number of alignment matches per chromosome and total length of the matches for the alignment between nuclear assembly (UMD3.1.1 and ARS-UCD1.2) and standard mitogenome linearization using LAST.

Table S4. List of all NUMT regions detected in the UMD_3.1.1 and ARS-UCD1.2 genome assemblies using LAST.

Table S5. Summary of identified NUMT regions per chromosome.

Table S6. Information on NUMT regions identified on the ARS-UCD1.2 assembly using LAST compared with NUMT regions found using BLASTN with default parameters.

Table S7. Information on NUMT regions identified on the ARS-UCD1.2 Assembly using LAST compared with NUMT regions found using consolidated results of all BLAST searches.

Table S8. Repetitive sequences in NUMT regions per class and family.

Table S9. NUMT regions located within gene boundaries.

Figure S1. Dot plots of potentially highly modified NUMT regions.

Figure S2. Alignment between NUMT regions ARS_10.7 and ARS_10.8, including 500 bp up- and downstream of

each region. Sequence from ARS-UCD1.2 assembly was used, and repetitive sequences were marked in gray.

Figure S3. Alignment between NUMT regions ARS_18.6, ARS_18.7and ARS_18.8, including 500 bp up- and downstream of each region. Sequence from ARS-UCD1.2 assembly was used, and repetitive sequences were marked in gray.

Figure S4. PCR amplification of 29 NUMT regions with genomic DNA from L1 Dominette 01449.

Figure S5. PCR amplification of 12 NUMT regions with genomic DNA from Dominette and other 7 breeds in a total of 17 animals.

Figure S6. Alignment of sequences obtained for amplicon UMD_15.B. The consensus sequence is 100% identical to the ARS-UCD1.2 genome assembly reference sequence (chromosome 15, positions 6050827-6051676).

AUB, Aubrac; BLA, Blonde d'Aquitaine; HOL, Holstein; MON, Montbéliarde, NOR, Normande

F and R indicate the forward and reverse DNA strand, respectively, while the + and – symbols indicate that the DNA sequence is aligned in the same orientation (+) or in the reverse-complementary orientation (-).

Table S1. NUMT regions selected for validation.

NUMT regions			Samples information			Amplicon length (bp)	Forward primer 5'-3' - Position and Sequence [data from UMD3.1.1 assembly]	Reverse Primer 5'-3' - Position and Sequence [data from UMD3.1.1 assembly]
CHR[1]	Identification	Length (bp)	Dominette	Animals	Breeds[3]			
28	UMD_28.2	396	yes	17	8	718	2048957 AGGTGGGTAACGTGGCTGAGAT 2048978	2049674 CATGTCCGAGGCCACACGGT 2049655
1	UMD_1.5	384	yes	17	8	749	113122818 GTGGAACTGAAGGGGCATGGCT 113122839	113123566 GCTCAGAGAAATGGGATGTCGTGGA 113123542
27	UMD_27.1	326	yes	17	8	783	4524499 AAATCCGTGCAGGGGTGGGG 4524518	4525281 GGCTTCCGGAGGACTGCTGG 4525262
14	UMD_14.1	363	yes	17	8	784	7337216 TTCTGCCCACAGCAACCCACT 7337236	7337999 ACACTTACTGCACCTGACGTGAC 7337977
8	UMD_8.6	547	yes	17	8	823	97773677 TCCGGGTGTCCAGATGAGGCT 97773697	97774499 ACAGTGCTCAGTGTGGGGGC 97774480
2	UMD_2.5	343	yes	17	8	825	6415171 CACAACCACCATCCCCACTAA 6415191	6415995 TGGTGGGTTTGGAGGAGTAGGTA 6415973
10	UMD_10.8	350	yes	17	8	899	51465075 ACTACATAACAATGCACACCACAC 51465098	51465976 GCATCAAAAGTGCCCAGGTTC 51465956
15	UMD.15.B[2]	334	yes	17	8	900	6297509 TGGGGAGACCCAGAATTGCC 6297528	6298408 ACCAAAGCCTACTTCCTATCTTCA 6298385
15	UMD_15.7	103	yes	17	8	900	68838983 CTCTCGATCGTTGTAGCATCT 68839003	68839882 GGCAAGGTTTTGCATTTATTTACCA 68839858
11	UMD_11.3	531	yes	17	8	1851	58229105 GAATGGAAGGCTTTCACCAGGCT 58229127	58230955 GGCCTATCTGCTCATCACCACCAAA 58230931
20	UMD_20.1	1383	yes	1	1	2065	6503632 ACAGTCCAGACTCCACATACTCTTT 6503656	6505696 GGTTGGCACATTGTATGTCAGCCA 6505673
18	UMD_18.9	1272	yes	17	8	2565	60175352 GCGAAACATTCTCTACTTACACAGTTCAG 60175380	60177916 ATCAGGGGTCCCGAAGGGGG 60177897
4	UMD_4.5	1141	yes	17	8	2665	37508716 AACGGCATTTCAGCATGTGGAGG 37508738	37511380 TGTGGACATCTCTGCTGATGATTACG 37511355
2	UMD_2.4	1613	yes	1	1	2812	4728910 ACCAATCCAATGTGGAGGCCCTT 4728932	4731721 CCTCTGCAAAAATGCTTCCTCCTCA 4731697
24	UMD_24.7	1883	yes	1	1	3170	53676714 TGAGCAGAGGAAGATTTCAGTTCA 53676737	53679883 TTCCGTATAATCCTGTGGTCAAA 53679861
8	UMD_8.4	1852	yes	1	1	3353	55007160 TTGCCCTACTCCTCATCCCCAGGA 55007183	55010512 AGATGGTCCAAGCCCAGACACCA 55010490
6	UMD_6.3	2727	yes	1	1	3779	72653070 TCCCTTGACTCCAAAAGGGACCA 72653092	72656848 GAGTTGGGGGAAATTAGAGGGAGGT 72656824
3	UMD_3.1	3007	yes	1	1	3781	91386327 TCAGAACAGCTGGAGGCTTG 91386346	91390107 CTATGTAAGTACAGTAGCCTGAGT 91390084
15	UMD_15.2	1752	yes	1	1	4268	33729281 TCAGAACCCCCAGAAGTTGC 33729300	33733548 AGTGTTAGATTTGAATTGTGGGATT 33733524
18	UMD_18.4	3048	yes	1	1	4353	39742211 CCGGGGAACGCCCCTGTAACC 39742231	39746563 AGCTGAGTCCCAGCATCCTGCTCAA 39746539
5	UMD_5.1	4135	yes	1	1	4426	17541799 TGGGAAGCCCCTAATTCAAACTAC 17541822	17546224 TTCGTGGCCAGAGGTCAATGTG 17546203
24	UMD_24.6	2712	yes	1	1	4476	44095212 CCATCGCTGGGGCTTTCCCACAT 44095234	44099687 ACTGGCCAACTGCAGGGATGC 44099667
X	UMD_X.2	1368	yes	1	1	4695	41218405 AAGGGAAACTGGGTGGGGTCCAT 41218427	41223099 TGCCGTGCTCCTATACAGTCAAATG 41223075
2	UMD_2.3	4903	yes	1	1	5744	3313398 ATCTGTGGACCTGGGGAGTCCAG 3313420	3319141 GCGGGCTCCAGAAGACTCTCCAT 3319119
10	UMD_10.1	4537	yes	1	1	7200	4971655 AGCCACACAACCACACGTCAT 4971675	4978854 GTTGGGTGTGGTGCTCACTGACT 4978832
12	UMD_12.4	1301	yes	1	1	8114	37060839 CCAAGGGAAACTGCGTCCAT 37060858	37068952 GGGGATCCACAGAGCATATACAG 37068930
4	UMD_4.9	7999	yes	1	1	8452	99612052 CTCGCTCCCCGCAAGTGGGA 99612071	99620503 GCCCATAATGCCCTGCCAAATCCCC 99620479
10	UMD_10.10	10457	yes	1	1	13162	63957687 CTTGTCTGTGTGGCTTCTATGAGGA 63957711	63970814 GGCACAAGTGGTCACTGCTGGTA 63970792
2	UMD_2.11	9632	yes	1	1	13274	90490992 TGTCAGCCTGGAAAACTCATGCAA 90491015	90504265 AGGCAACTCACAGTAGCACCAGG 90504243

1 CHR was used for chromosome.

2 NUMT region detected using BLAST on UMD3.1.1 during preliminary study (personal data). Location: CHR15:6297989-6298323 on UMD3.1.1.

3 Breeds: Hereford (1), Montbéliarde (3), Holstein (3), Normande (2), Limousine (1), Salers (2), Blonde d'Aquitaine (3, and Aubrac (2). Number of samples between brackets.

Table S2. Estimation of the impact of *e*-value thresholds for the alignments between nuclear assembly (UMD3.1.1 and ARS-UCD1.2) and standard mitogenome linearization linearization using LAST.

e-value Filter[1]	Alignment matches information[2]					
	Number[3]	Length (kbp)[4]	Mitochondrial (kbp)[5]	Nuclear (kbp)[6]	Gaps (kbp)[7]	Identity (%)[8]
$< 10^{-2}$	434<br>446	242.1<br>245.4	240.3<br>243.7	236.9<br>240.0	2.0<br>2.1	77.9%<br>78.2%
$< 10^{-3}$	429<br>441	241.8<br>245.1	240.1<br>243.4	236.6<br>239.7	2.0<br>2.1	77.9%<br>78.2%
$< 10^{-4}$	426<br>439	241.5<br>244.9	239.8<br>243.2	236.3<br>239.6	2.0<br>2.1	77.9%<br>78.2%
$< 10^{-5}$	417<br>431	240.7<br>244.3	239.0<br>242.6	235.5<br>239.0	2.0<br>2.1	77.9%<br>78.2%

1 e-value filter used to select the alignments to be analyzed.
2 Within each cell, values on top refer to assembly ARS-UCD1.2 and values on bottom refer to assembly UMD3.1.1.
3 Number of alignment matches detected.
4 Total length of alignment matches.
5 Total length of mitochondrial genome aligned.
6 Total length of nuclear genome aligned.
7 Total length of alignment gaps.
8 Identity between mitochondrial and NUMT regions sequences.

Table S3. Number of alignment matches per chromosome and total length of the matches for the alignment between nuclear assembly (UMD3.1.1 and ARS-UCD1.2) and the standard mitogenome linearization using LAST.

Chromosome	UMD3.1.1		ARS-UCD1.2		Differences between the results of both assemblies			
	Number of matches	Total length (kbp)[1]	Number of matches	Total length (kbp)[1]	Number of matches		Length (kbp)	
1	31	17,397	31	17,397	0	0%	0	0%
2	33	23,292	32	23,453	1	3%	161	1%
3	5	3,869	5	3,869	0	0%	0	0%
4	26	9,207	26	9,479	0	0%	272	3%
5	8	4,682	8	4,682	0	0%	0	0%
6	4	3,318	4	3,318	0	0%	0	0%
7	7	1,460	7	1,460	0	0%	0	0%
8	6	3,014	6	3,014	0	0%	0	0%
9	11	10,469	10	11,356	1	9%	887	8%
10	52	26,206	50	26,230	2	4%	24	0%
11	25	12,443	25	13,077	0	0%	634	5%
12	8	2,298	7	1,779	1	13%	519	23%
13	13	3,502	14	3,743	1	8%	241	7%
14	9	3,128	9	3,128	0	0%	0	0%
15	16	7,542	17	7,777	1	6%	235	3%
16	11	9,192	9	12,912	2	18%	3,720	40%
17	19	9,926	18	8,843	1	5%	1,083	11%
18	14	4,044	13	3,340	1	7%	704	17%
19	4	1,954	3	626	1	25%	1,328	68%
20	13	4,657	13	4,658	0	0%	1	0%
21	3	444	3	444	0	0%	0	0%
22	35	23,800	34	22,699	1	3%	1,101	5%
23	-	-	-	-	-	-	-	-
24	15	8,664	13	9,134	2	13%	470	5%
25	1	491	-	-	1	100%	491	100%
26	12	5,274	11	5,080	1	8%	194	4%
27	3	577	3	577	0	0%	0	0%
28	34	19,754	33	19,289	1	3%	465	2%
29	4	1,069	4	1,069	0	0%	0	0%
X	15	17,126	9	8,444	6	40%	8,682	51%
Unplaced[2]	2	792	9	5,383	7	350%	4,591	580%
**Total**	**439**	**239,591**	**426**	**236,260**	**13**	**3%**	**3,331**	**1%**

1 The "total length" was calculated by summing the length of the alignment matches, per chromosome.
2 Unplaced scaffolds. These alignments were not used for the NUMT region detection.

Table S4. List of all NUMT regions detected in the UMD_3.1.1 and ARS-UCD1.2 genome assemblies using LAST.

UMD_3.1.1					ARS-UCD1.2				
NUMT region	CHR[1]	Start position	End position	Length (bp)[2]	NUMT region	CHR[1]	Start position	End position	Length (bp)[2]
UMD_1.1	1	12811430	12820694	9265	ARS_1.1	1	13401385	13410648	9264
UMD_1.2	1	32469929	32470337	409	ARS_1.2	1	32903076	32903484	409
UMD_1.3	1	74649203	74649317	115	ARS_1.3	1	74038942	74039056	115
UMD_1.4	1	95764899	95764975	77	ARS_1.4	1	94998201	94998277	77
UMD_1.5	1	113123100	113123483	384	ARS_1.5	1	112219471	112219854	384
UMD_1.6	1	115885591	115885645	55	ARS_1.6	1	114959465	114959519	55
UMD_1.7	1	117471891	117496262	24372	ARS_1.7	1	116533243	116557613	24371
UMD_1.8	1	122191272	122193709	2438	ARS_1.8	1	121223372	121225803	2432
UMD_1.9	1	133413306	133413746	441	ARS_1.9	1	132309962	132310402	441
UMD_1.10	1	138756116	138758547	2432	ARS_1.10	1	156921322	156923753	2432
UMD_2.1	2	1357968	1358718	751	ARS_2.1	2	1427787	1428537	751
UMD_2.2	2	2015595	2030298	14704	ARS_2.2	2	2085710	2100413	14704
UMD_2.3	2	3313866	3318768	4903	ARS_2.3	2	3386219	3391182	4964
UMD_2.4	2	4729846	4731458	1613	ARS_2.4	2	4797610	4799222	1613
UMD_2.5	2	6415574	6415916	343	ARS_2.5	2	6480665	6481007	343
UMD_2.6	2	22686429	22686934	506	ARS_2.6	2	22650482	22650987	506
UMD_2.7	2	40740974	40741257	284	ARS_2.7	2	40600460	40600743	284
UMD_2.8	2	52774170	52774309	140	ARS_2.8	2	52673849	52673988	140
UMD_2.9	2	67011643	67011700	58	ARS_2.9	2	66659803	66659860	58
UMD_2.10	2	85335357	85336095	739	ARS_2.10	2	84940388	84941126	739
UMD_2.11	2	90493431	90503062	9632	ARS_2.11	2	90077160	90086791	9632
UMD_2.12	2	104605082	104605197	116	ARS_2.12	2	104083158	104083273	116
UMD_2.13	2	107186195	107186386	192	ARS_2.13	2	106463510	106463701	192
UMD_2.14	2	129504072	129504239	168	ARS_2.14	2	128905747	128905914	168
UMD_2.15	2	136691052	136691229	178	ARS_2.15	2	136110007	136110184	178
UMD_3.1	3	91386578	91389584	3007	ARS_3.1	3	90809110	90812116	3007
UMD_3.2	3	99796429	99796564	136	ARS_3.2	3	99198342	99198477	136
UMD_3.3	3	99828551	99828872	322	ARS_3.3	3	99230430	99230751	322
UMD_3.4	3	99862510	99862837	328	ARS_3.4	3	99264028	99264355	328
UMD_3.5	3	103779161	103779236	76	ARS_3.5	3	103184780	103184855	76
UMD_4.1	4	5284020	5284709	690	ARS_4.1	4	5366685	5367374	690
UMD_4.2	4	19690600	19690716	117	ARS_4.2	4	19758601	19758717	117
UMD_4.3	4	24277775	24291164	13390	ARS_4.3	4	24333569	24346570	13002
UMD_4.4	4	24601057	24601170	114	ARS_4.4	4	24652762	24652875	114
UMD_4.5	4	37508943	37510083	1141	ARS_4.5	4	37325762	37326902	1141
UMD_4.6	4	42683450	42683517	68	ARS_4.6	4	42448079	42448146	68
UMD_4.7	4	56686497	56687568	1072	ARS_4.7	4	56374325	56375395	1071
UMD_4.8	4	80975436	80975612	177	ARS_4.8	4	80267172	80267348	177
UMD_4.9	4	99612082	99620080	7999	ARS_4.9	4	98710770	98718689	7920

UMD_4.10	4	104872550	104872620	71	ARS_4.10	4	104035179	104035249	71
UMD_4.11	4	120003757	120003811	55	ARS_4.11	4	119203491	119203545	55
UMD_5.1	5	17541842	17545976	4135	ARS_5.1	5	17464784	17468918	4135
UMD_5.2	5	43409831	43410003	173	ARS_5.2	5	43182930	43183102	173
UMD_5.3	5	85264122	85268921	4800	ARS_5.3	5	84845707	84850507	4801
UMD_6.1	6	35551068	35551463	396	ARS_6.1	6	34113156	34113551	396
UMD_6.2	6	66521568	66521661	94	ARS_6.2	6	64877892	64877985	94
UMD_6.3	6	72653617	72656343	2727	ARS_6.3	6	70953991	70956717	2727
UMD_6.4	6	107492381	107492481	101	ARS_6.4	6	104868249	104868349	101
UMD_7.1	7	44153787	44156091	2305	ARS_7.1	7	42715220	42717524	2305
UMD_7.2	7	45968958	45973186	4229	ARS_7.2	7	44347242	44351470	4229
UMD_7.3	7	88353467	88353764	298	ARS_7.3	7	86001941	86002238	298
UMD_7.4	7	89248312	89248475	164	ARS_7.4	7	86894023	86894186	164
UMD_8.1	8	12388363	12388710	348	ARS_8.1	8	12480290	12480637	348
UMD_8.2	8	22028929	22028995	67	ARS_8.2	8	21996578	21996644	67
UMD_8.3	8	43163618	43163741	124	ARS_8.3	8	42880668	42880791	124
UMD_8.4	8	55007988	55009839	1852	ARS_8.4	8	54721070	54722921	1852
UMD_8.5	8	66314774	66314849	76	ARS_8.5	8	65836070	65836145	76
UMD_8.6	8	97773915	97774461	547	ARS_8.6	8	96101997	96102543	547
UMD_9.1	9	4693464	4704097	10634	ARS_9.1	9	4620016	4631438	11423
UMD_9.2	9	21020735	21020830	96	ARS_9.2	9	20771910	20772005	96
UMD_9.3	9	30950148	30950302	155	ARS_9.3	9	30557292	30557446	155
UMD_9.4	9	87842916	87843164	249	ARS_9.4	9	86696116	86696365	250
UMD_9.5	9	89091682	89091957	276	ARS_9.5	9	87933259	87933534	276
UMD_10.1	10	4973487	4978023	4537	ARS_10.1	10	5035087	5039122	4036
UMD_10.2	10	11212244	11212475	232	ARS_10.2	10	11272763	11272994	232
UMD_10.3	10	19790980	19791487	508	ARS_10.3	10	19956795	19957302	508
UMD_10.4	10	44933995	44952004	18010	ARS_10.4	10	44886476	44904484	18009
UMD_10.5	10	47869493	47871116	1624	ARS_10.5	10	47816150	47817773	1624
UMD_10.6	10	50586732	50592503	5772	ARS_10.6	10	50516649	50522420	5772
UMD_10.7	10	50963865	50964197	333	ARS_10.7	10	50901742	50902074	333
UMD_10.8	10	51465531	51465880	350	ARS_10.8	10	51403811	51404160	350
UMD_10.9	10	55795335	55801211	5877	ARS_10.9	10	55715617	55721487	5871
UMD_10.10	10	63958493	63968949	10457	ARS_10.10	10	63725820	63736276	10457
UMD_10.11	10	65676073	65681035	4963	ARS_10.11	10	65448422	65453384	4963
UMD_10.12	10	65735813	65736354	542	ARS_10.12	10	65508161	65508702	542
UMD_10.13	10	69298230	69298799	570	ARS_10.13	10	69055010	69055662	653
UMD_10.14	10	82440863	82440933	71	ARS_10.14	10	82074954	82075024	71
UMD_11.1	11	6127471	6127540	70	ARS_11.1	11	6096889	6096958	70
UMD_11.2	11	45647014	45675229	28216	ARS_11.2	11	45785205	45814229	29025
UMD_11.3	11	58229679	58230209	531	ARS_11.3	11	58306700	58307230	531
UMD_11.4	11	66128143	66128308	166	ARS_11.4	11	66151403	66151568	166
UMD_12.1	12	1297272	1297488	217	ARS_12.1	12	1361751	1361967	217
UMD_12.2	12	14909867	14909937	71	ARS_12.2	12	14863282	14863352	71
UMD_12.3	12	21537809	21537951	143	ARS_12.3	12	21466683	21466825	143
UMD_12.4	12	37062275	37063575	1301	ARS_12.4	12	36803416	36804716	1301

UMD_12.5	12	44437284	44437499	216	ARS_12.5	12	44140180	44140395	216
UMD_12.6	12	48951951	48952017	67	ARS_12.6	12	48646882	48646948	67
UMD_12.7	12	91161677	91162195	519	-	-	-	-	-
UMD_13.1	13	11651632	11661620	9989	ARS_13.1	13	11595615	11607830	12216
UMD_13.2	13	42209273	42209430	158	ARS_13.2	13	41843382	41843539	158
UMD_13.3	13	47449835	47449946	112	ARS_13.3	13	47091490	47091601	112
UMD_13.4	13	54093728	54093913	186	ARS_13.4	13	53620969	53621154	186
UMD_13.5	13	54109271	54109473	203	ARS_13.5	13	53636512	53636714	203
UMD_13.6	13	54121640	54121752	113	ARS_13.6	13	53648881	53648993	113
UMD_14.1	14	7337446	7337808	363	ARS_14.1	14	6297951	6298313	363
UMD_14.2	14	20558273	20559126	854	ARS_14.2	14	18948380	18949233	854
UMD_14.3	14	20574557	20576908	2352	ARS_14.3	14	18964070	18966421	2352
UMD_14.4	14	31524626	31525035	410	ARS_14.4	14	29829010	29829419	410
UMD_14.5	14	41687095	41688956	1862	ARS_14.5	14	39548096	39549957	1862
UMD_15.1	15	5525096	5525335	240	ARS_15.1	15	5284235	5284474	240
UMD_15.2	15	33730018	33731769	1752	ARS_15.2	15	33195471	33197222	1752
UMD_15.3	15	42340458	42340537	80	ARS_15.3	15	41775778	41775857	80
UMD_15.4	15	43500972	43502691	1720	ARS_15.4	15	42909504	42911223	1720
UMD_15.5	15	48377742	48378262	521	ARS_15.5	15	47709837	47710357	521
UMD_15.6	15	57413543	57418838	5296	ARS_15.6	15	56630034	56635329	5296
UMD_15.7	15	68839014	68839116	103	ARS_15.7	15	68011884	68011986	103
-	-	-	-	-	ARS_15.8	15	83957438	83957672	235
UMD_16.1	16	619485	627782	8298	ARS_16.1	16	817986	829794	11809
UMD_16.2	16	11534531	11534736	206	ARS_16.2	16	10919931	10920136	206
UMD_16.3	16	35580599	35580890	292	ARS_16.3	16	34799081	34799372	292
UMD_16.4	16	39494946	39501585	6640	ARS_16.4	16	38638713	38645352	6640
UMD_16.5	16	71896700	71896821	122	ARS_16.5	16	70006735	70006856	122
UMD_16.6	16	81557560	81557701	142	ARS_16.6	16	79837757	79837898	142
UMD_17.1	17	121482	123436	1955	ARS_17.1	17	143331	143787	457
UMD_17.2	17	44439910	44441071	1162	ARS_17.2	17	5546577	5546915	339
UMD_17.3	17	58913228	58926699	13472	ARS_17.3	17	56657147	56670617	13471
UMD_17.4	17	75066179	75066305	127	ARS_17.4	17	73028819	73028945	127
UMD_18.1	18	14608737	14608829	93	ARS_18.1	18	14566459	14566551	93
UMD_18.2	18	18842220	18842923	704	-	-	-	-	-
UMD_18.3	18	27844259	27844840	582	ARS_18.3	18	27752696	27753277	582
UMD_18.4	18	39743537	39746584	3048	ARS_18.4	18	39607935	39610982	3048
UMD_18.5	18	41972024	41972103	80	ARS_18.5	18	41828736	41828815	80
UMD_18.6	18	59834657	59835166	510	ARS_18.6	18	59412752	59413261	510
UMD_18.7	18	60053758	60054260	503	ARS_18.7	18	59762045	59762547	503
UMD_18.8	18	60063121	60063463	343	ARS_18.8	18	59807938	59808280	343
UMD_18.9	18	60176031	60177302	1272	ARS_18.9	18	59867553	59868824	1272
UMD_19.1	19	2967720	2967907	188	ARS_19.1	19	2865085	2865272	188
UMD_19.2	19	6198111	6198238	128	ARS_19.2	19	5991750	5991877	128
UMD_19.3	19	10964054	10964363	310	ARS_19.3	19	10734497	10734806	310
UMD_19.4	19	22814079	22815406	1328	-	-	-	-	-
UMD_20.1	20	6503671	6505053	1383	ARS_20.1	20	6585524	6586906	1383

UMD_20.2	20	8450412	8461589	11178	ARS_20.2	20	8528431	8539609	11179
UMD_20.3	20	19880742	19880998	257	ARS_20.3	20	19873588	19873844	257
UMD_21.1	21	15119501	15119789	289	ARS_21.1	21	14761903	14762191	289
UMD_21.2	21	29334663	29334717	55	ARS_21.2	21	28798166	28798220	55
UMD_21.3	21	34241992	34242091	100	ARS_21.3	21	33850846	33850945	100
UMD_22.1	22	6960754	6995583	34830	ARS_22.1	22	6888148	6922983	34836
UMD_22.2	22	19705582	19711621	6040	ARS_22.2	22	19625034	19629899	4866
UMD_22.3	22	47697196	47709002	11807	ARS_22.3	22	47138697	47150504	11808
UMD_24.1	24	2303106	2303250	145	-	-	-	-	-
UMD_24.2	24	12837964	12838115	152	ARS_24.2	24	12538399	12538550	152
UMD_24.3	24	14068674	14068760	87	ARS_24.3	24	13764585	13764671	87
UMD_24.4	24	36397041	36399885	2845	ARS_24.4	24	35993080	35995923	2844
UMD_24.5	24	36805023	36805084	62	ARS_24.5	24	36401697	36401758	62
UMD_24.6	24	44095810	44098521	2712	ARS_24.6	24	43654538	43657378	2841
UMD_24.7	24	53677566	53679448	1883	ARS_24.7	24	53207391	53209273	1883
UMD_24.8	24	61511586	61514197	2612	ARS_24.8	24	61001514	61003946	2433
UMD_25.1	25	2602431	2602921	491	-	-	-	-	-
UMD_26.1	26	21566895	21566962	68	ARS_26.1	26	21698927	21698994	68
UMD_26.2	26	25886672	25886745	74	ARS_26.2	26	25659797	25659870	74
UMD_26.3	26	28229853	28230043	191	ARS_26.3	26	28050711	28050901	191
UMD_26.4	26	28312962	28313152	191	-	-	-	-	-
UMD_26.5	26	32034952	32044769	9818	ARS_26.5	26	31752082	31761878	9797
UMD_27.1	27	4524836	4525161	326	ARS_27.1	27	5656129	5656454	326
UMD_27.2	27	15492252	15492396	145	ARS_27.2	27	16435739	16435883	145
UMD_27.3	27	36791828	36791933	106	ARS_27.3	27	37106412	37106517	106
UMD_28.1	28	2049061	2049456	396	ARS_28.1	28	492427	492573	147
UMD_28.2	28	2372583	2373248	666	ARS_28.2	28	825313	825708	396
UMD_28.3	28	27793309	27794291	983	ARS_28.3	28	27634869	27 635 851	983
UMD_28.4	28	35554699	35579985	25287	ARS_28.4	28	35355283	35379236	23954
UMD_28.5	28	39174119	39174270	152	ARS_28.5	28	38944686	38944837	152
UMD_28.6	28	42163395	42176287	12893	ARS_28.6	28	41830981	41843872	12892
UMD_29.1	29	328507	333938	5432	ARS_29.1	29	354897	360137	5241
UMD_29.2	29	14652127	14652248	122	ARS_29.2	29	14565176	14565297	122
UMD_29.3	29	35601868	35601956	89	ARS_29.3	29	35020663	35020751	89
UMD_X.1	X	27385987	27386087	101	ARS_X.1	X	27407737	27407837	101
UMD_X.2	X	41219983	41221350	1368	ARS_X.2	X	38546369	38546701	333
UMD_X.3	X	58182947	58184078	1132	-	-	-	-	-
UMD_X.4	X	80170353	80176879	6527	ARS_X.4	X	75132748	75139274	6527
UMD_X.5	X	110578228	110578333	106	ARS_X.5	X	105099177	105099282	106
UMD_X.6	X	114169713	114173028	3316	ARS_X.6	X	108948384	108948571	188
UMD_X.7	X	114458829	114462899	4071	-	-	-	-	-
UMD_X.8	X	118057033	118057287	255	ARS_X.8	X	112806246	112806500	255
UMD_X.9	X	118082230	118083259	1030	ARS_X.9	X	112831022	112832051	1030

1 The abbreviation "CHR" was used for chromosome.
2 The start and end positions of the NUMT regions in the nuclear genome were used to calculate the length.

Note: in most cases, it was possible to correlate NUMT regions from both assemblies. However in some cases it is difficult or not possible to decide if the NUMT region was present in both assemblies.

Table S5. Summary of identified NUMT regions per chromosome.

Chromosome	UMD_3.1.1 assembly		ARS-UCD1.2 assembly	
	Number of NUMT regions	Total length[1] (bp)	Number of NUMT regions	Total length[1] (bp)
1	10	39,988	10	39,980
2	15	34,327	15	34,388
3	5	3,869	5	3,869
4	11	24,894	11	24,426
5	3	9,108	3	9,109
6	4	3,318	4	3,318
7	4	6,996	4	6,996
8	6	3,014	6	3,014
9	5	11,410	5	12,200
10	14	53,846	14	53,421
11	4	28,983	4	29,792
12	7	2,534	6	2,015
13	6	10,761	6	12,988
14	5	5,841	5	5,841
15	7	9,712	8	9,947
16	6	15,700	6	19,211
17	4	16,716	4	14,394
18	9	7,135	8	6,431
19	4	1,954	3	626
20	3	12,818	3	12,819
21	3	444	3	444
22	3	52,677	3	51,510
23	-	-	-	-
24	8	10,498	7	10,302
25	1	491	-	-
26	5	10,342	4	10,130
27	3	577	3	577
28	6	40,377	6	38,524
29	3	5,643	3	5,452
X	9	17,906	7	8,540
Total	173	441,879	166	430,264

1 The" total length" was calculated by summing the length of the NUMT regions present in each chromosome. The start and end positions of the NUMT regions in the nuclear genome were used to calculate the length of each region. The length of each region can be found in Table S4.

Table S6. Information on NUMT regions identified on the ARS-UCD1.2 assembly using LAST compared with NUMT regions found using BLASTN with default parameters.

NUMT regions, identified using LAST, that do not overlap with the ones identified using BLASTN.				
NUMT region	CHR	Start position	End position	Length (bp)
ARS_1.3	1	74038942	74039056	115
ARS_1.4	1	94998201	94998277	77
ARS_2.8	2	52673849	52673988	140
ARS_2.12	2	104083158	104083273	116
ARS_3.2	3	99198342	99198477	136
ARS_3.3	3	99230430	99230751	322
ARS_3.4	3	99264028	99264355	328
ARS_4.11	4	119203491	119203545	55
ARS_8.1	8	12480290	12480637	348
ARS_8.2	8	21996578	21996644	67
ARS_12.1	12	1361751	1361967	217
ARS_12.6	12	48646882	48646948	67
ARS_13.3	13	47091490	47091601	112
ARS_15.8	15	83957438	83957672	235
ARS_16.6	16	79837757	79837898	142
ARS_17.1	17	143331	143787	457
ARS_18.1	18	14566459	14566551	93
ARS_19.1	19	2865085	2865272	188
ARS_26.2	26	25659797	25659870	74
ARS_27.3	27	37106412	37106517	106
ARS_28.1	28	492427	492573	147
ARS_29.3	29	35020663	35020751	89
-	-	-	-	-
-	-	-	-	-
-	-	-	-	-
-	-	-	-	-

NUMT regions, identified using BLASTN, that do not overlap with the ones identified using LAST.				
NUMT region	CHR	Start position	End position	Length (bp)
-	1	127662613	127662655	43
-	2	74826206	74826488	283
-	7	110679201	110679786	586
-	8	113066625	113067210	586
-	9	8320570	8320680	111
-	9	12710035	12710830	796
-	11	62480940	62481233	294
-	14	19556538	19556600	63
-	14	28049025	28049189	165
-	15	6051307	6051622	316
-	15	83956479	83957122	644
-	16	79837058	79837651	594
-	17	26423806	26423875	70
-	18	5067400	5067431	32
-	18	14566629	14566815	187
-	18	59008564	59008764	201
-	18	59201573	59201732	160
-	18	59256340	59256499	160
-	18	59319315	59319589	275
-	18	59848143	59849408	1,266
-	19	4409807	4409960	154
-	20	51849409	51849479	71
-	21	52512649	52512733	85
-	23	37282226	37282509	284
-	24	13629712	13629910	199
-	24	48386502	48386568	67

Table S7. Information on NUMT regions identified on the ARS-UCD1.2 Assembly using LAST compared with NUMT regions found using consolidated results of all BLAST searches.

NUMT regions, identified using LAST, that do not overlap with the ones identified using BLAST searches.				
NUMT region	CHR	Start position	End position	Length (bp)
ARS_1.3	1	74038942	74039056	115
ARS_2.12	2	104083158	104083273	116
ARS_3.2	3	99198342	99198477	136
ARS_3.3	3	99230430	99230751	322
ARS_3.4	3	99264028	99264355	328
ARS_4.11	4	119203491	119203545	55
ARS_8.2	8	21996578	21996644	67
ARS_12.1	12	1361751	1361967	217
ARS_12.6	12	48646882	48646948	67
ARS_13.3	13	47091490	47091601	112
ARS_19.1	19	2865085	2865272	188
ARS_27.3	27	37106412	37106517	106
ARS_28.1	28	492427	492573	147
ARS_29.3	29	35020663	35020751	89
-	-	-	-	-
-	-	-	-	-
-	-	-	-	-
-	-	-	-	-
-	-	-	-	-
-	-	-	-	-
-	-	-	-	-
-	-	-	-	-
-	-	-	-	-
-	-	-	-	-
-	-	-	-	-
-	-	-	-	-
-	-	-	-	-
-	-	-	-	-
-	-	-	-	-
-	-	-	-	-
-	-	-	-	-
-	-	-	-	-
-	-	-	-	-
-	-	-	-	-
-	-	-	-	-
-	-	-	-	-
-	-	-	-	-
-	-	-	-	-
-	-	-	-	-

NUMT regions, identified using BLAST searches, that do not overlap with the ones identified using LAST.				
NUMT region	CHR	Start position	End position	Length (bp)
-	1	97119345	97119990	646
-	1	127662613	127662655	43
-	2	74826186	74826488	303
-	3	37679787	37679854	68
-	3	99284969	99285184	216
-	6	68426646	68427263	618
-	7	71900898	71901084	187
-	7	110678938	110681611	2,674
-	8	157571	157741	171
-	8	98095287	98095479	193
-	8	113066362	113069035	2,674
-	9	8320570	8320680	111
-	9	12709990	12711060	1071
-	10	41467721	41467914	194
-	11	62480940	62481233	294
-	12	1899398	1899565	168
-	12	66290589	66290953	365
-	14	19556538	19556744	207
-	14	28049025	28049240	216
-	15	6051288	6051667	380
-	15	46547435	46547546	112
-	15	56699713	56700183	471
-	16	421778	421962	185
-	17	26423806	26423875	70
-	18	5067400	5067431	32
-	18	8768554	8768740	187
-	18	58726970	58727230	261
-	18	58843915	58844146	232
-	18	58879312	58879543	232
-	18	59008564	59008803	240
-	18	59077117	59077348	232
-	18	59201515	59201782	268
-	18	59256307	59256551	245
-	18	59319315	59319589	275
-	18	59796238	59796496	259
-	18	59838284	59838542	259
-	18	59848090	59849408	1,319
-	19	4409807	4409970	164
-	20	51849352	51849498	147

-	-	-	-	-
-	-	-	-	-
-	-	-	-	-
-	-	-	-	-
-	-	-	-	-
-	-	-	-	-
-	-	-	-	-
-	-	-	-	-
-	-	-	-	-
-	-	-	-	-
-	-	-	-	-
-	-	-	-	-
-	-	-	-	-
-	-	-	-	-
-	-	-	-	-
-	-	-	-	-
-	-	-	-	-

-	20	62160537	62160867	331
-	21	52512538	52512824	287
-	23	1117213	1117408	196
-	23	37282226	37282521	296
-	24	3330842	3331128	287
-	24	13629712	13629910	199
-	24	48386502	48386568	67
-	25	27515136	27515234	99
-	26	2832229	2832401	173
-	26	33015098	33015270	173
-	26	46166014	46166392	379
-	27	37093433	37094081	649
-	28	484082	486228	2,147
-	28	511309	513450	2,142
-	X	116470822	116471410	589
-	X	120905188	120905562	375
-	X	136773121	136773299	179

Table S8. Repetitive sequences in NUMT regions per class and family.[1]

Repetitive elements class and family[1]	Flanking region 1kb upstream			NUMT Regions			Flanking region 1kb downstream		
	Number[3]	Overlap length (pb)[4]	%	Number[3]	Overlap length (bp)[4]	%	Number[3]	Overlap length (bp)[4]	%
**DNA**	**21**	**4,105**	**5,3%**	**5**	**356**	**0,2%**	**23**	**3,810**	**4,7%**
hAT	1	186	0,2%	4	311	0,2%	1	107	0,1%
TcMar-Mariner	0	0	0,0%	1	45	0,0%	3	100	0,1%
hAT-Ac	0	0	0,0%	0	0	0,0%	1	68	0,1%
hAT-Blackjack	1	52	0,1%	0	0	0,0%	1	98	0,1%
hAT-Charlie	13	2,645	3,4%	0	0	0,0%	14	2,711	3,3%
hAT-Tip100	3	827	1,1%	0	0	0,0%	1	423	0,5%
TcMar-Tigger	3	395	0,5%	0	0	0,0%	2	303	0,4%
**LINE**	**154**	**41,845**	**54,4%**	**220**	**104,062**	**64,7%**	**175**	**45,936**	**56,2%**
L1	69	18,626	24,2%	72	41,947	26,1%	88	21,759	26,6%
L2	19	3,347	4,4%	1	1	0,0%	15	2,575	3,2%
RTE-BovB	66	19,872	25,9%	147	62,114	38,6%	69	21,343	26,1%
CR1	0	0	0,0%	0	0	0,0%	2	199	0,2%
Penelope	0	0	0,0%	0	0	0,0%	1	60	0,1%
**Low_complexity**	**8**	**377**	**0,5%**	**6**	**274**	**0,2%**	**5**	**206**	**0,3%**
Low_complexity	8	377	0,5%	6	274	0,2%	5	206	0,3%
**LTR**	**38**	**9,198**	**12,0%**	**39**	**6,157**	**3,8%**	**33**	**7,055**	**8,6%**
ERV1	12	4,285	5,6%	9	2,639	1,6%	14	3,257	4,0%
ERVK	11	1,629	2,1%	30	3,518	2,2%	8	822	1,0%
ERVL	9	1,771	2,3%	0	0	0,0%	4	1,230	1,5%
ERVL-MaLR	6	1,513	2,0%	0	0	0,0%	7	1,746	2,1%
**Satellite**	2	746	**1,0%**	0	0	**0,0%**	0	0	**0,0%**
centr	2	746	1,0%	0	0	0,0%	0	0	0,0%
**Simple_repeat**	**34**	**1,213**	**1,6%**	**60**	**2,176**	**1,4%**	**41**	**1,544**	**1,9%**
Simple_repeat	34	1,213	1,6%	60	2,176	1,4%	41	1,544	1,9%
**SINE**	**138**	**19,137**	**24,9%**	**302**	**44,303**	**27,6%**	**164**	**22,958**	**28,1%**
Core-RTE	26	4,538	5,9%	72	13,985	8,7%	33	5,855	7,2%
MIR	18	2,227	2,9%	2	206	0,1%	17	1,877	2,3%
tRNA	16	2,206	2,9%	26	4,221	2,6%	18	2,603	3,2%
tRNA-Core-RTE	78	10,166	13,2%	202	25,891	16,1%	96	12,623	15,4%
**snRNA**	0	0	**0,0%**	**1**	**107**	**0,1%**	0	0	**0,0%**
snRNA	0	0	0,0%	1	107	0,1%	0	0	0,0%
**tRNA**	0	0	**0,0%**	**48**	**3,300**	**2,1%**	**3**	**207**	**0,3%**
tRNA	0	0	0,0%	48	3,300	2,1%	3	207	0,3%
**Other**	**3**	**249**	**0,3%**	**0**	**0**	**0,0%**	0	0	**0,0%**
Other	3	249	0,3%	0	0	0,0%	0	0	0,0%
**Total**	**398**	**76,870**	**100,0%**	**681**	**160,735**	**100,0%**	**456**	**81,716**	**100,0%**

1 Repetitive sequence's position and classification were retrieved from the database BovineMine v1.6 (http://128.206.116.13:8080/bovinemine/begin.do).
2 Family and/or class not specified or unknown.
3 Number of repetitive sequences.
4 Overlap between the repetitive sequence position and the NUMT region position.

Table S9. NUMT regions located within gene boundaries.

Gene stable ID or NUMT region ID	CHR[1]	Gene or NUMT region start	Gene or NUMT region end	Size (bp)	Strand	Gene description
ENSBTAG00000018994	1	94 985 031	95 001 222	16192	1	TNF superfamily member 10 [Source:VGNC Symbol;Acc:VGNC:36172]
ARS_1.4	1	94 998 201	94 998 277	77		
ENSBTAG00000049521	1	116 535 664	116 535 939	276	-1	
ARS_1.7	1	116 533 243	116 557 613	24371		
ENSBTAG00000003833	1	132 298 308	132 334 597	36290	-1	solute carrier family 35 member G2 [Source:VGNC Symbol;Acc:VGNC:34837]
ARS_1.9	1	132 309 962	132 310 402	441		
ENSBTAG00000052593	2	2 097 072	2 097 178	107	1	
ARS_2.2	2	2 085 710	2 100 413	14704		
ENSBTAG00000049463	2	3 387 277	3 387 540	264	1	
ARS_2.3	2	3 386 219	3 391 182	4964		
ENSBTAG00000018795	2	6 460 095	6 592 355	132261	-1	PMS1 homolog 1, mismatch repair system component [VGNC;Acc:VGNC:33077]
ARS_2.5	2	6 480 665	6 481 007	343		
ENSBTAG00000006970	2	22 528 224	22 693 132	164909	1	Obg like ATPase 1 [Source:VGNC Symbol;Acc:VGNC:32416]
ARS_2.6	2	22 650 482	22 650 987	506		
ENSBTAG00000001132	2	52 388 547	52 825 730	437184	1	glycosyltransferase like domain containing 1 [Source:VGNC Symbol;Acc:VGNC:29690]
ARS_2.8	2	52 673 849	52 673 988	140		
ENSBTAG00000050589	2	52 561 468	52 794 962	233495	-1	
ARS_2.8	2	52 673 849	52 673 988	140		
ENSBTAG00000020696	2	84 741 175	85 088 822	347648	-1	HECT, C2 and WW domain containing E3 ligase 2 [VGNC; Acc:VGNC:56116]
ARS_2.10	2	84 940 388	84 941 126	739		
ENSBTAG00000017436	2	90 045 394	90 168 893	123500	-1	C2 calcium dependent domain containing 6 [Source:VGNC Symbol;Acc:VGNC:26637]
ARS_2.11	2	90 077 160	90 086 791	9632		
ENSBTAG00000010413	2	104 067 181	104 128 350	61170	1	transmembrane protein 169 [Source:VGNC Symbol;Acc:VGNC:35994]
ARS_2.12	2	104 083 158	104 083 273	116		
ENSBTAG00000006821	2	104 068 554	104 116 082	47529	-1	peroxisomal trans-2-enoyl-CoA reductase [Source:VGNC Symbol;Acc:VGNC:32738]
ARS_2.12	2	104 083 158	104 083 273	116		

ENSBTAG00000016572	2	106 447 346	106 532 901	85556	-1	ubiquitin specific peptidase 37 [Source:VGNC Symbol;Acc:VGNC:36726]
ARS_2.13	2	106 463 510	106 463 701	192		
ENSBTAG00000000229	3	99 242 275	99 264 955	22681	1	cytochrome P450 4A25-like [Source:NCBI gene;Acc:784417]
ARS_3.4	3	99 264 028	99 264 355	328		
ENSBTAG00000020869	4	5 336 436	5 441 308	104873	1	dopa decarboxylase [Source:VGNC Symbol;Acc:VGNC:55748]
ARS_4.1	4	5 366 685	5 367 374	690		
ENSBTAG00000007680	4	19 514 202	19 990 379	476178	-1	thrombospondin type 1 domain containing 7A [Source:VGNC Symbol;Acc:VGNC:35850]
ARS_4.2	4	19 758 601	19 758 717	117		
ENSBTAG00000015073	4	24 500 692	24 921 062	420371	-1	isoprenoid synthase domain containing [Source:VGNC Symbol;Acc:VGNC:30303]
ARS_4.4	4	24 652 762	24 652 875	114		
ENSBTAG00000021182	4	56 119 633	56 598 768	479136	1	dedicator of cytokinesis 4 [Source:VGNC Symbol;Acc:VGNC:28158]
ARS_4.7	4	56 374 325	56 375 395	1071		
ENSBTAG00000032121	4	80 159 127	80 928 936	769810	-1	succinyl-CoA:glutarate-CoA transferase [Source:VGNC Symbol;Acc:VGNC:35462]
ARS_4.8	4	80 267 172	80 267 348	177		
ENSBTAG00000013976	4	98 689 853	98 789 432	99580	1	ATP/GTP binding protein like 3 [Source:VGNC Symbol;Acc:VGNC:25722]
ARS_4.9	4	98 710 770	98 718 689	7920		
ENSBTAG00000021761	4	104 027 627	104 194 547	166921	-1	B-Raf proto-oncogene, serine/threonine kinase [Source:VGNC Symbol;Acc:VGNC:26551]
ARS_4.10	4	104 035 179	104 035 249	71		
ENSBTAG00000004958	4	118 732 679	119 258 101	525423	-1	protein tyrosine phosphatase, receptor type N2 [Source:NCBI gene;Acc:616515]
ARS_4.11	4	119 203 491	119 203 545	55		
ENSBTAG00000003748	5	43 134 292	43 266 434	132143	-1	CCR4-NOT transcription complex subunit 2 [Source:VGNC Symbol;Acc:VGNC:27517]
ARS_5.2	5	43 182 930	43 183 102	173		
ENSBTAG00000002972	5	84 818 340	84 887 228	68889	1	cancer susceptibility 1 [Source:NCBI gene;Acc:526444]
ARS_5.3	5	84 845 707	84 850 507	4801		
ENSBTAG00000019808	6	33 504 739	34 501 208	996470	-1	coiled-coil serine rich protein 1 [Source:VGNC Symbol;Acc:VGNC:26993]
ARS_6.1	6	34 113 156	34 113 551	396		
ENSBTAG00000011817	6	64 820 535	64 960 787	140253	-1	gamma-aminobutyric acid type A receptor alpha2 subunit [NCBI gene;Acc:282236]
ARS_6.2	6	64 877 892	64 877 985	94		
ENSBTAG00000044044	6	70 874 084	70 978 580	104497	-1	clock circadian regulator [Source:VGNC Symbol;Acc:VGNC:27456]
ARS_6.3	6	70 953 991	70 956 717	2727		

ENSBTAG00000003652	6	104 853 328	104 868 645	15318	1	transmembrane protein 128 [Source:VGNC Symbol;Acc:VGNC:35961]
ARS_6.4	6	104 868 249	104 868 349	101		
ENSBTAG00000054972	9	4 621 622	4 621 918	297	-1	
ARS_9.1	9	4 620 016	4 631 438	11423		
ENSBTAG00000052141	9	4 628 627	4 633 122	4496	-1	
ARS_9.1	9	4 620 016	4 631 438	11423		
ENSBTAG00000007388	9	86 692 238	86 726 552	34315	-1	zinc finger CCCH-type containing 12D [Source:VGNC Symbol;Acc:VGNC:37100]
ARS_9.4	9	86 696 116	86 696 365	250		
ENSBTAG00000009679	9	87 781 650	87 966 376	184727	1	pleckstrin homology and RhoGEF domain containing G1 [VGNC;Acc:VGNC:33014]
ARS_9.5	9	87 933 259	87 933 534	276		
ENSBTAG00000034998	10	5 026 117	5 109 495	83379	-1	SUMO interacting motifs containing 1 [Source:VGNC Symbol;Acc:VGNC:54482]
ARS_10.1	10	5 035 087	5 039 122	4036		
ENSBTAG00000004990	10	19 888 399	20 126 828	238430	1	neogenin 1 [Source:VGNC Symbol;Acc:VGNC:32007]
ARS_10.3	10	19 956 795	19 957 302	508		
ENSBTAG00000021945	10	44 846 568	44 939 145	92578	-1	nidogen 2 [Source:VGNC Symbol;Acc:VGNC:32074]
ARS_10.4	10	44 886 476	44 904 484	18009		
ENSBTAG00000025522	10	55 636 874	56 319 410	682537	-1	unc-13 homolog C [Source:VGNC Symbol;Acc:VGNC:36665]
ARS_10.9	10	55 715 617	55 721 487	5871		
ENSBTAG00000030881	10	69 005 149	69 075 633	70485	1	transmembrane protein 260 [Source:VGNC Symbol;Acc:VGNC:36069]
ARS_10.13	10	69 055 010	69 055 662	653		
ENSBTAG00000025898	11	5 978 170	6 119 371	141202	-1	TBC1 domain family member 8 [Source:VGNC Symbol;Acc:VGNC:35645]
ARS_11.1	11	6 096 889	6 096 958	70		
ENSBTAG00000019134	12	21 443 115	21 543 353	100239	-1	NIMA related kinase 5 [Source:VGNC Symbol;Acc:VGNC:31996]
ARS_12.3	12	21 466 683	21 466 825	143		
ENSBTAG00000008647	12	43 998 992	44 525 321	526330	-1	kelch like family member 1 [Source:VGNC Symbol;Acc:VGNC:53821]
ARS_12.5	12	44 140 180	44 140 395	216		
ENSBTAG00000044007	12	48 526 712	48 803 497	276786	-1	Kruppel like factor 12 [Source:VGNC Symbol;Acc:VGNC:30628]
ARS_12.6	12	48 646 882	48 646 948	67		
ENSBTAG00000003955	15	56 532 192	56 637 170	104979	1	myosin VIIA [Source:VGNC Symbol;Acc:VGNC:31830]
ARS_15.6	15	56 630 034	56 635 329	5296		

ENSBTAG00000049875	16	818 146	818 352	207	-1	
ARS_16.1	16	817 986	829 794	11809		
ENSBTAG00000048925	16	819 140	819 436	297	-1	
ARS_16.1	16	817 986	829 794	11809		
ENSBTAG00000050274	16	820 704	820 967	264	-1	
ARS_16.1	16	817 986	829 794	11809		
ENSBTAG00000033056	16	34 429 413	34 853 620	424208	1	phospholipase D family member 5 [Source:VGNC Symbol;Acc:VGNC:32999]
ARS_16.3	16	34 799 081	34 799 372	292		
ENSBTAG00000052192	18	14 557 133	14 566 670	9538	1	spermatogenesis associated 33 [Source:NCBI gene;Acc:100848931]
ARS_18.1	18	14 566 459	14 566 551	93		
ENSBTAG00000049640	18	59 391 349	59 414 228	22880	-1	
ARS_18.6	18	59 412 752	59 413 261	510		
ENSBTAG00000011623	19	10 674 179	10 802 801	128623	1	vacuole membrane protein 1 [Source:VGNC Symbol;Acc:VGNC:36806]
ARS_19.3	19	10 734 497	10 734 806	310		
ENSBTAG00000007651	21	28 759 374	28 803 585	44212	1	threonyl-tRNA synthetase like 2 [Source:VGNC Symbol;Acc:VGNC:35606]
ARS_21.2	21	28 798 166	28 798 220	55		
ENSBTAG00000010027	22	47 133 914	47 158 681	24768	1	choline dehydrogenase [Source:VGNC Symbol;Acc:VGNC:27285]
ARS_22.3	22	47 138 697	47 150 504	11808		
ENSBTAG00000004612	26	25 203 123	26 181 296	978174	1	sortilin related VPS10 domain containing receptor 3 [Source:NCBI gene;Acc:531405]
ARS_26.2	26	25 659 797	25 659 870	74		
ENSBTAG00000054529	26	31 755 445	31 755 732	288	1	
ARS_26.5	26	31 752 082	31 761 878	9797		
ENSBTAG00000011032	27	5 446 148	5 686 242	240095	1	microcephalin 1 [Source:VGNC Symbol;Acc:VGNC:31321]
ARS_27.1	27	5 656 129	5 656 454	326		
ENSBTAG00000020657	27	16 420 193	16 546 078	125886	-1	FAT atypical cadherin 1 [Source:VGNC Symbol;Acc:VGNC:52771]
ARS_27.2	27	16 435 739	16 435 883	145		
ENSBTAG00000021497	28	27 570 723	27 963 222	392500	1	cadherin related 23 [Source:NCBI gene;Acc:784716]
ARS_28.3	28	27 634 869	27 635 851	983		
ENSBTAG00000004280	X	105 052 662	105 194 351	141690	1	sushi repeat containing protein X-linked [Source:VGNC Symbol;Acc:VGNC:50570]
ARS_X.5	X	105 099 177	105 099 282	106		

1 CHR was used for chromosome.

Figure S1. Dot plots of potentially highly modified NUMT regions.[1]

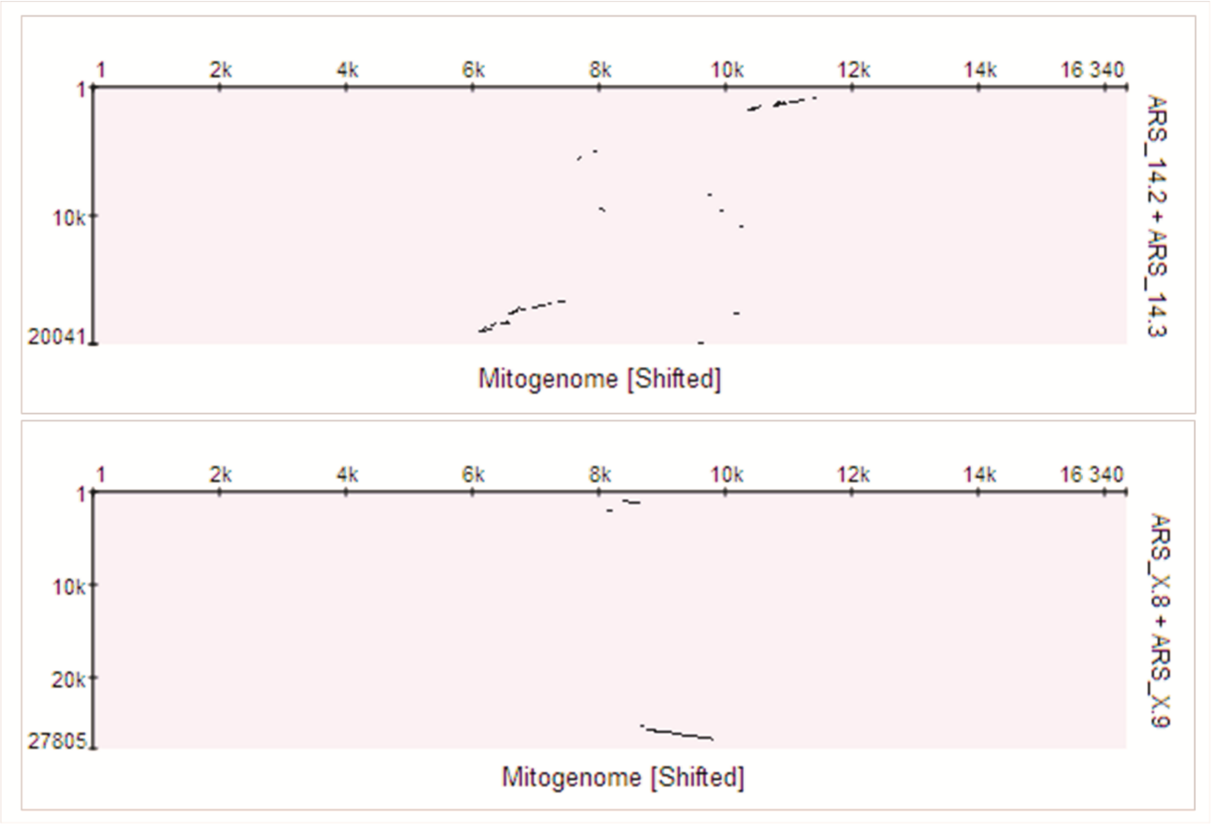

1 Examples are based on results obtained from NUMTs discovered with the ARS_UCD1.2 genome assembly. Mitochondrial DNA sequence is plotted on X axis and NUMT regions are plotted on Y axis. The positions indicated in the axes of the dot plots start at 1 and go to the complete length of each sequence. Therefore, dot plot representations are not in the same scale for the Y axis and the position of the shifted representation of the mitochondrial DNA is not adjusted for linearization cut-point.

Figure S2. Alignment between NUMT regions ARS_10.7 and ARS_10.8, including 500 bp up- and downstream of each region. Sequence from ARS-UCD1.2 assembly was used, and repetitive sequences were marked in gray.[1]

```
ARS_10.7  --TTTCAGTTCTGTTCAACAACAAAAAAGAAATGACAATACCTACTACATAACAATGCAC
ARS_10.8  GTTTTCAGTTCTGTTCAACAACAAAAAAGAAATGACAATACCTACTACATAACAATGCAC
            **********************************************************

ARS_10.7  ACCACACACATGATACATTATACAGTACCATGCTAAGCCACATTGAAGTGAGGCAAAGAA
ARS_10.8  ACCACACACATGATACATTATACAGTACCGTGCTAAGCCACATTGAAGTGAGGCAAAGAA
          ***************************** ******************************

ARS_10.7  AAATCAAGACTACCAAAAAAATAAATAAATAAAAATCAAGACTACCAATCCTGTAGTTGT
ARS_10.8  AAATCAAGCCTACCAAAAAAATAAATAAATAAAAATCAAGACTACCAATCCTGTAGTT--
          ******** *************************************************

ARS_10.7  TGAAATTTTTGATATTTTGTTCATCACTGATTTTTGGTGTTAATTGTCAATTTTCAAAAT
ARS_10.8  -GAAATTTTTGATATTTTGTTCATCACTGATTTTTGGTGTTAATTGTCAATTTTCAAAGT
           ********************************************************* *

ARS_10.7  ATTGCATTAAAATTATATTTATCTTCATTACTGAGTGTTTGGGGCACCCCCTTAAATTTC
ARS_10.8  ACTACATTAAAATTATATTTATCTTCATTACTGAGTGTTTGGGGCACCCCCTTAAATTTC
          * * ********************************************************

ARS_10.7  ACACTTAAGTGCTTTACCTCACATGGCCCTATGTAGCTCTGATGCTATAATAGCCAGTG
ARS_10.8  ACACTTAAGTGCTTTACCTCACGTGGCCCTATCTAGCTCTGATGCTATAATAGCCAGTG
          ********************** ********* ***************************

ARS_10.7  TTTATGTATCAGGCTCTAGGCTAAATGCTTTACCTATAGTATTTGATTTATTCCTTGCAA
ARS_10.8  TTTATGTATCAGGCTCTAGGCTAAATGCTTTACCTATAGTATTTGATTTATTCCTTGCAA
          ************************************************************

ARS_10.7  CAATCCATGAATAAAACTGAGACTTAAAGACATTACTAAGTCTTGTCCAAGTCCCAAAGC
ARS_10.8  CAATCCATGAATAAAACTGAGACTTAAAGACATTACTAAGTCTTGTCCAAGTCCCAAAGC
          ************************************************************

ARS_10.7  TGTTTAGCACTAGAA-GAACAAATACACAGATGAAGAATATGGAGGCTCTGTTTGCATGT
ARS_10.8  TGTTTAGCAGTAGAAAGAACAAATACCCAGATAAAGAATACGGAGGCTCTGTTTGCATGT
          ********* ***** ********** ***** ******* *******************

ARS_10.7  AAGTTTCAGATAATTCATCTGTAGCTCATGGGCAGATAGGAGTCGTGGCTGAAAAGGTAG
ARS_10.8  AAGTTTCAGATAATTCATCTGTAGCTCGTGGGCAGATAGGAGTCGTGGCTGAAAAGGTAG
          *************************** ********************************

ARS_10.7  TTACTGT---GTCTGATGTTTA--------GACTCCTGCTGGGATTTGCAATGTTAGGCA
ARS_10.8  TTACTGTCTGATGTAGAGTATAACTAGAAAGACTCCTGCTGGGATTTGCAATATTAGGCA
          *******    * *   ** **        ********************** *******

ARS_10.7  GATTTCTAACAGTGGGAATTTCATCATGATAAGATATTTGACAGGGCTCGTAGATCAATA
ARS_10.8  TATTTCTAACAGTGGGAATTTCATCATGATAAGATATTTGACAGGGCTTGTAGATCAATA
           *********************************************** ***********

ARS_10.7  AATTAATTGGCAATAATCTTGATTCGTGGGTGGATTTTCAGAGGTTGGTATTTGATGTT
ARS_10.8  AACTAATTGGCAATAATCTTGATTCGTGGGTGGATTTTCAGAGGTTGGTATTTGATGTT
          ** *********************************************************

ARS_10.7   TCTGTATTTGAAATATAATGACAATTTTTCATGTTATTGGCCCTAGTCAGATTTCATGTA
ARS_10.8   TCTGTATTTGAAATATAATGACAATTTTTCATGTTATTGGCCCTAGTCAGATATCATGTA
           *************************************************** ********

ARS_10.7   AAAATAGTAACAGAGAAAACGAAATAGATACAGTATAACTTTTAAGTACTATTTGTGTGG
ARS_10.8   AAAATAGTAATAGAGAAAACGAAATAGATACAGTATAAATTTTAAGTACTATTTGTGTGG
           ********** *************************** *********************

ARS_10.7   AATTTGAAGTACTATATGGTGGAATTTGAACCTGGGTACTTCTGATGCCACGGCCTATTT
ARS_10.8   AATTTGAAGTACTATATAGTGGAATTTGAACCTGGGCACTTTTGATGCCATGGCCTGTTT
           ***************** ****************** **** ******** ***** ***
```

```
ARS_10.7   TTTAACCAGATGATGCTGGATGGCCTTCCGATGACCACATCAGTCTGGGAGGGAACAATG
ARS_10.8   TTTAACCAGATGATGCTGGATGGCCTTCCGATGACCACATCAGTCTGGGAGAGAACAATG
           *************************************************** ********

ARS_10.7   CATCACAAACTTCAGTACATAAAGCATGTGGAAACATGAATTGTGAATAAATGCAGTTTT
ARS_10.8   CATCACAAACTTCAGTACATAGAGCGTGTGGAAACGTGAATTGTGAATAAATGCTGTTTT
           ********************* *** ********* ****************** *****

ARS_10.7   TGTTCCCCTTTTCTTCCCCCAAGGATTGCACCTATCAGAGAATATGAAGAGTAACATATA
ARS_10.8   TGTTCCCCTTTTCTTCCCCCAAGGATTGCACCTATCAGAGAATATGAAGAGTAACATATA
           ************************************************************

ARS_10.7   AAAAATTTTATTTGGGGGAGAGCAAATTAATTTTAACACTCTGATTTCTT----------
ARS_10.8   AAAAATTTTATTTGGGGGAGAGCAAATTAATTTTAACACTCTGATTTCTTTTTTTTTTTT
           **************************************************

ARS_10.7   ------------------ACCCCTTCACCCAGGTCTACCCTGGAAAAGAGTGAAGATTTA
ARS_10.8   TTAACACTCTGATTTCTTACCCCTTCACCCAGGTCTACACTGGAAAAGAATGAAGATTTA
                             ******************** ********** **********

ARS_10.7   GTGTCATTTTCCAAATGAACCTGATAAAAGCATCTTTAGTTTGATATACAAAGAACCAAA
ARS_10.8   GTGTCATTTTCCAAATGAACCTGATAAAAGCATCTTTAGTTTGATATACAAAGAGCCAAA
           ****************************************************** *****

ARS_10.7   AGCATTTTGGGATTTGAGCACCTTCTTAAATAATGAAACTGAACCAAGAGCTTT
ARS_10.8   AGCATTTTGGGATTTGAGCACCTTCTTAAATA----------------------
           ********************************
```

1 Assembly sequence was recovered from NCBI with masked sequences in lower case letters. This masking was used as a reference to mark repetitive regions above.

Figure S3. Alignment between NUMT regions ARS_18.6, ARS_18.7and ARS_18.8, including 500 bp up- and downstream of each region. Sequence from ARS-UCD1.2 assembly was used, and repetitive sequences were marked in gray.

```
ARS_18.7   ---------ACACATACACCATTCATAGGGCTCATCCAGAGGCCGAAACTTGCCTCCTCT
ARS_18.8   ---------ACACATACACCATTCATAGGGCTCATCCAGAGGCCGAAACTTGCCTCCTCT
ARS_18.6   TTGCAACACACGCATACACCATTCATAGGGCTCATCCAGAGGCCGAAACCTGCCTCCTCT
                    ** ************************************* **********

ARS_18.7   CACAACTCTCCTAGACCCCCAATAGCTTCCTCTCCACTTTCCCACATGTTTTCACCACTT
ARS_18.8   CACAACTCTCCTAGACCCCCAATAGCTTCCTCTCCACTTTCCCACATGTTTTCACCACTT
ARS_18.6   CACAACTCTCCTAGACCCCTGATAGCTTCCTCTCCGCTTTCCCACATGTTTTCACCACTT
           *******************  ************** ************************

ARS_18.7   TTCTGTGCTCTGTTGAGCAACAGGGCTAAACAGCAGAGACCACTGGGCCTGGGCAACACA
ARS_18.8   TTCTGTGCTCTGTTGAGCAACAGGGCTAAACAGCAGAGACCACTGGGCCTGGGCAACACA
ARS_18.6   TTCTGTGCTCTATTGAGCAACAGGGCTAAACAGTAGAGACCACGGGGCCTGGGCAACACA
           *********** ********************* ********* ****************

ARS_18.7   CAGAAAACACACAAGCCGCAAATGCAAGGGCACTGGGCACTCAGTATGGAGACCAAGAAA
ARS_18.8   CAGAAAACACACAAGCCGCAAATGCAAGAGCACTGGGCACTCAGTATGGAGACCAAGAAA
ARS_18.6   CA-AAAACACACAAGCCACAAATGCAAGGGCACTGGGCACTCAGTATGGAGACCAAGAAA
           ** ************** ********** *******************************

ARS_18.7   CTGGCCCTCTTGGGAAAACATGCCAGGAACTGG-GATATCACGAAGTTTTAAAGGTGTTT
ARS_18.8   CTGGCCCTCTTGGGAAAACATACCAGGAACTGG-GATATCACGAAGTTTTAAAGGTGTTT
ARS_18.6   CTGGCCCTCCTGGGAAAGCATGCCAGGAACTGGAGATATCACAAAGTTTTAAAGGTCCTT
           ********* ******* *** *********** ******** *************  **

ARS_18.7   GAGATTTGGCTCGACTGAGCTCAGTGAAGAAGTCACAGAAAAAGGCCCCAATCTCACTGC
ARS_18.8   GAGATTTAGCTCGACTGAGCTCAGTGAAGAAGTCACAGAAAAAGGCCCCAATCTCACTGC
ARS_18.6   GAGATTTGGCTCGATTGAGCTCAGTGAAGAAGTCTCAGAGAAAGGCCCCAATCTCACTGC
           ******* ****** ******************* **** ********************

ARS_18.7   CGCCTGTTGTACCTGACTGACCTAATTGGAAGATACCCCAGCTCTTTAAGGAAGCGGGGC
ARS_18.8   CGCCTGTTGTACCTGACTGACCTAACTGGAAGATACCCCAGCTCTTTAAGGAAGCGGGGC
ARS_18.6   CGCCTGTTGTCTCTGACTGACCTAACTGGAAGATACCCCAGCTCTTTAAAGAAGCGGGGC
           **********  ************* *********************** **********

ARS_18.7   CTCCTTCATAGCCCCTTAGATGCCAAAGCTTCTGGCAACTCTAACCACAGTAAAATCAGT
ARS_18.8   CTCCTTCATAGCCCCTTAGATGCCAAAGCTTCTGGCAACTCTAACCACAGTAAAATCAGT
ARS_18.6   CTCCTTCATAGCCCCTTAGATTCCAAAGCTTCTGGC---------CACAGTAAAACCAGT
           ********************* **************         ********** ****

ARS_18.7   ATCCCCCACTCTGAGTAGATAAACTGCTGAAAGCAATGTGTTTCCCCACTACAGCCCTCA
ARS_18.8   ATCCCCCACTCTGAGTAGATAAACTGCTGAAAGCAATGTGTTTCCCCACTACAGCCCTCA
ARS_18.6   ATCCCCCACTCTGAGTAGATAAACCGCTGAAAGCAATGAGTTTCCCTACTACAGCCCTCA
           ************************ ************* ******* *************

ARS_18.7   TTATGAAGTCTCCCATTAACAGGATTACCAATCCTCACAGGCTTCAATTCTTAAGATCTT
ARS_18.8   TTATGAAGTCTCCCATTAACAGGATTACCAATCCTCACAGGCTTCAATTCTTAAGATCTT
ARS_18.6   TTATGAAGTCTCCCATTAACAGGATTACCAATCCTCACAGGCTTCCATTCTTAAGATCTT
           ********************************************* **************

ARS_18.7   ATTATCCAATCAGCCCACATGTTGCACACCAAGACCAGAGCCT--------CTGTCTCTA
ARS_18.8   ATTATCCAATCAGCCCACATGTTGCACACCAAGACCAGAGCCT--------CTGTCTCTA
ARS_18.6   ATTATCCAATCAGCCCACATGTTGCACACCAAGACCTGAGCCTATTAATAACCCTGATTA
           ************************************ ******        *  *   **

ARS_18.7   CACCCACACAGCTGTGTACAGCACTCGTGTCACTGGCTTCACAATTCTAGGACAATCTCC
ARS_18.8   CACCCACACAGCTGTGTACAGCACTCGTGTCACTGGCTTCACAATTCTAGGACAACCTCC
ARS_18.6   CACCCACACAGCTGCGTACAGCACTGGTGTCACTGGCTTCACAATTCTAGGACAACCTCC
           ************** ********** ***************************** ****

ARS_18.7   CTTCTCCGATTAATTTTAATTAATGAAGACAACCCACTCCTACTCAACTCTATCAAAGGT
ARS_18.8   CTTCTCCGATTAATTTTAATTAATGAAGACAACCCACTCCTACTCAACTCTATCAAAGGT
ARS_18.6   CTTCTCCAGTTAATTTTAATTAATGAAAACAACCCACTCCTACTCAACTCTATCAAAGGT
            *******  ****************** ********************************
```

```
ARS_18.7   CTGTTAGCTAGAAGCATTTTGAACCAATTTTTT--ATCTCTGACAATATTACACCAACAA
ARS_18.8   CTGTTAGCTAGAAGCATTTTGAACCAATTTTTT--ATCTCTGACAATATTACACCAACAA
ARS_18.6   CTGTTAGCTAGAAGCATTTTCAACCAGTTTTTTTTATCTCTGACAATATTCCACCAACAA
           ******************** ***** ******  *************** *********

ARS_18.7   CATTGCCACAAATAAACAGGCCTCGTTACTTCAAGCAAACTGTTCCTTTTCCACCTGCCC
ARS_18.8   CATTGCCACAAATAAACAGGCCTCGTTACTTCAAGCAAACTGTTCCTTTTCCACCTGCCC
ARS_18.6   CATTCCCACAAATAACCAGGCCTCATTACTTGAAGCTAACTGCTCCTTGTCCACCTGCCC
           **** ********** ******** ****** **** ***** ***** ***********

ARS_18.7   CACATAATCATCTTAGGTTTAGTGTAGCACTGACAATCAACCTTAATGCACAAAATCTAA
ARS_18.8   CACATAATCATCTTAGGTTTAGTGTAGCACTGACAATCAACCTTAATGCACAAA-TCTAA
ARS_18.6   CACATAATCA---TAGGTTTAGTGTAGCACTGACAGTCAACCTTGATGCACAAAATCTAA
           **********   ********************** ******** ********* *****

ARS_18.7   AACTATCCCTCAACTGTCTTCAGATTCTCCGATCTCTTAAGATATTTCCCCACTATTATA
ARS_18.8   AACTATCCCTCAACTGTCTTCAGATTCTCCGATCTCTTAAGATATTTCCCCACTATTATA
ARS_18.6   AACTATCCCTCAAATGTCTTCAGATTCTCCGATCTCTTAAGATATTTCCCCACTATTATA
           ************* **********************************************

ARS_18.7   CACCACTTTCCATTGCACTTGTCAGCAATCCATAATTCAGCATTATCCACACTGGGTCTC
ARS_18.8   -ACCACTTTCCATTGCACTTGTCAGCAATCCATAATTCAGCATTATCCACACTGGGTCTC
ARS_18.6   CACCCCTTTCCACTGCACTTGTCAGCAGTCCATAATT-AGCATTATCCACACTGGGTCTC
            *** ******* ************** ********* **********************

ARS_18.7   ACTGACTGGAAAACTACCAACCAACGTTCACTTTTAAAATTCAAATAAAACACTAGTATC
ARS_18.8   ACTGACTGGAAAACTACCAACCAACATTCACTTTTAAAATTCAAATAAAACACTAGTATC
ARS_18.6   ACTGACTGGAAAACTACCAACCAAAACTCACTCCTTTAATTCAAATAAAACACTAGTATC
           ************************   *****  *  ***********************

ARS_18.7   AGACCAAAGAGGCTTCCCAGGTGGCTCAGTGGTAAAGAATCCTCCTTCCAATGAAGGAGA
ARS_18.8   AGACCAAAGAGGCTTCCCAGGTGGCTCAGTGGTAATGAATCCTCCTTCCAATGAAGGAGA
ARS_18.6   AGACCAAAGAGGCTTCCCAGGTGGCTCAGTGGTAAC-AATCCTCCTGCCAAT---GGAGA
           ***********************************  ********* *****   *****

ARS_18.7   TGCAAGAGATGGGGGTTTTATCCCTGAGTCAGAAGATCCACAGGATTAGGAAATGGCAGC
ARS_18.8   TGAAAGAGATGGGGGTTTTATCCCTGAGTCAGAAGATCCACAGGATTAGGAAATGGCAGC
ARS_18.6   TGCAAGAAATGGGGGTTTTATCCCTGAGTCAGAAGATCCCCAGGATTAGGAAATGGCAAC
           ** **** ******************************** ***************** *

ARS_18.7   CGACTCCAGTATTCTTGCCTGGGAATTCCCATGGACACAGCAGCCTGGAAGGCTACACTC
ARS_18.8   CCACTCCAGTATTCTTGCCTGGGAATTCCCATGGACACAGCAGCCTGGAAGGCTACACTC
ARS_18.6   CCACTCCAGTATTCTTGCCTGGGAAATCCCATGGACACAGGAGCCTGGCGGGCTACACTC
           * *********************** ************** ******* *  ********

ARS_18.7   CATGGGGTCTCA--GAGTCGGAAATGTCTGAGCATAGAGACCATTTTATATTTTTCTTTG
ARS_18.8   CATGGGGTCTCAAAGAGTCGGAAATGTCTGAGTATAGAGACCAT----------------
ARS_18.6   CATGGGGTCTCAAAGAGTCGGAAATGTCTGAGGATAGAGACCATTTTATATTTTTCTTTG
           ************  ****************** ***********

ARS_18.7   ATTCTAGAGCTCATATCAGTAACCAGCATCACTGAAAATTATCTCCTAAAATGAACAGTC
ARS_18.8   ------------------------------------------------------------
ARS_18.6   ATTCTGGAGCTCATACCAGTAACCAGCATCACTGAAAATAATCTCCTAAAGTGAAGAGCC


ARS_18.7   TCTC--GTCAATTTATTACCCTGGTCATATAAACCAGAAAAGGACAGTAATACGCCTCCC
ARS_18.8   ------------------------------------------------------------
ARS_18.6   TCTGTAGTCAATTTATTACCCTGGTCATATAAACCAGAAAAGGACAGTAATACGCCTCCC


ARS_18.7   TAACACTCGAGAAAGAACTCCAGCTC-
ARS_18.8   ---------------------------
ARS_18.6   TAACACTCGAGAAAGAACTTCAGCGCC
```

1 Assembly sequence was recovered from NCBI with masked sequences in lower case letters. This masking was used as a reference to mark repetitive regions above.

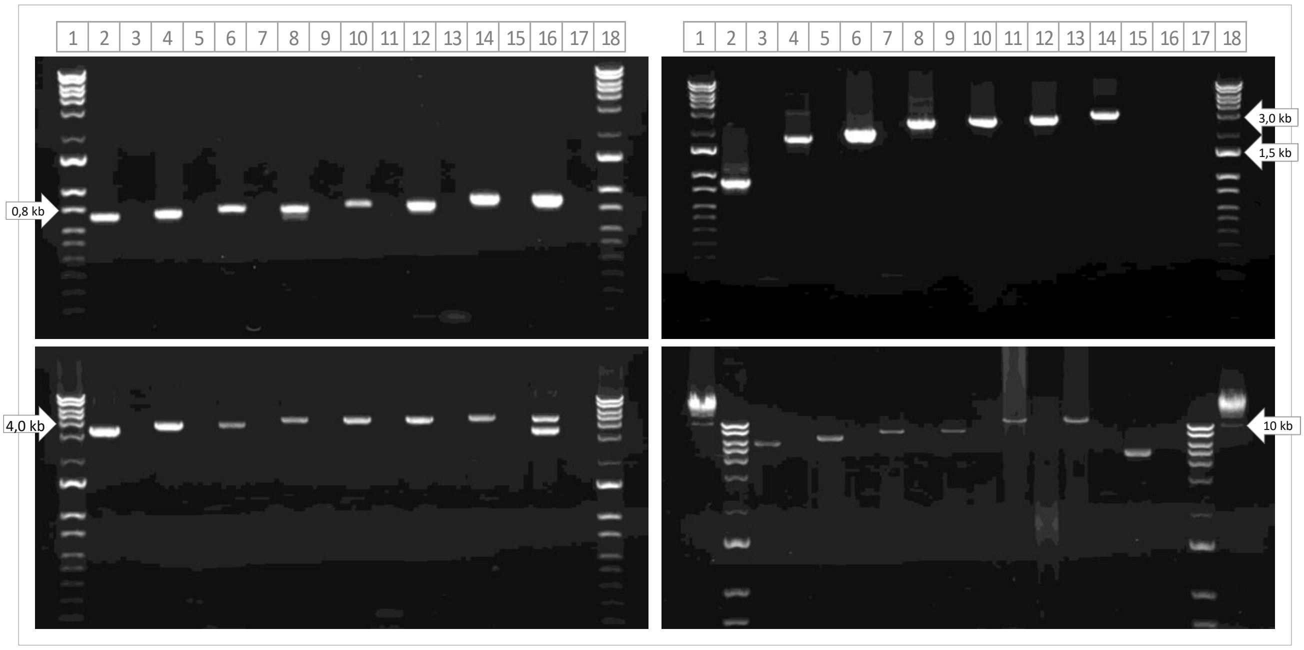

Figure S4. PCR amplification of 29 NUMT regions with genomic DNA from L1 Dominette 01449. On the right of each amplicon, the respective negative control was charged.

On the top-left gel, 8 NUMT regions, from left to right: UMD_28.2, UMD_1.5, UMD_27.1, UMD_14.1, UMD_8.6, UMD_ 2.5, UMD_10.8 and UMD_15.B.

On the top-right gel, 7 NUMT regions, from left to right: UMD_15.7, UMD_11.3, UMD_20.1, UMD_18.9, UMD_4.5, UMD_2.4 and UMD_24.7.

On the bottom-left gel, 8 NUMT regions, from left to right: UMD_8.4, UMD_6.3, UMD_3.1, UMD_15.2, UMD_18.4, UMD_5.1, UMD_24.6 and UMD_X.2.

On the bottom-right gel, 6 NUMT regions, from left to right: UMD_2.3, UMD_10.1, UMD_12.4, UMD_4.9, UMD_10.10 and UMD_2.11.

ExactLadder DNA PreMix 2 Log (Ozyme) was used in all gels (lanes 2 and 17 for the bottom-right gel and lanes 1 and 18 for the other three gels). For the bottom-right gel, was used an additional ladder on lanes 1 and 18 (Lambda DNA Mono Cut Mix from New England Biolabs) and a reference amplicon of 5 kb on lane 15.

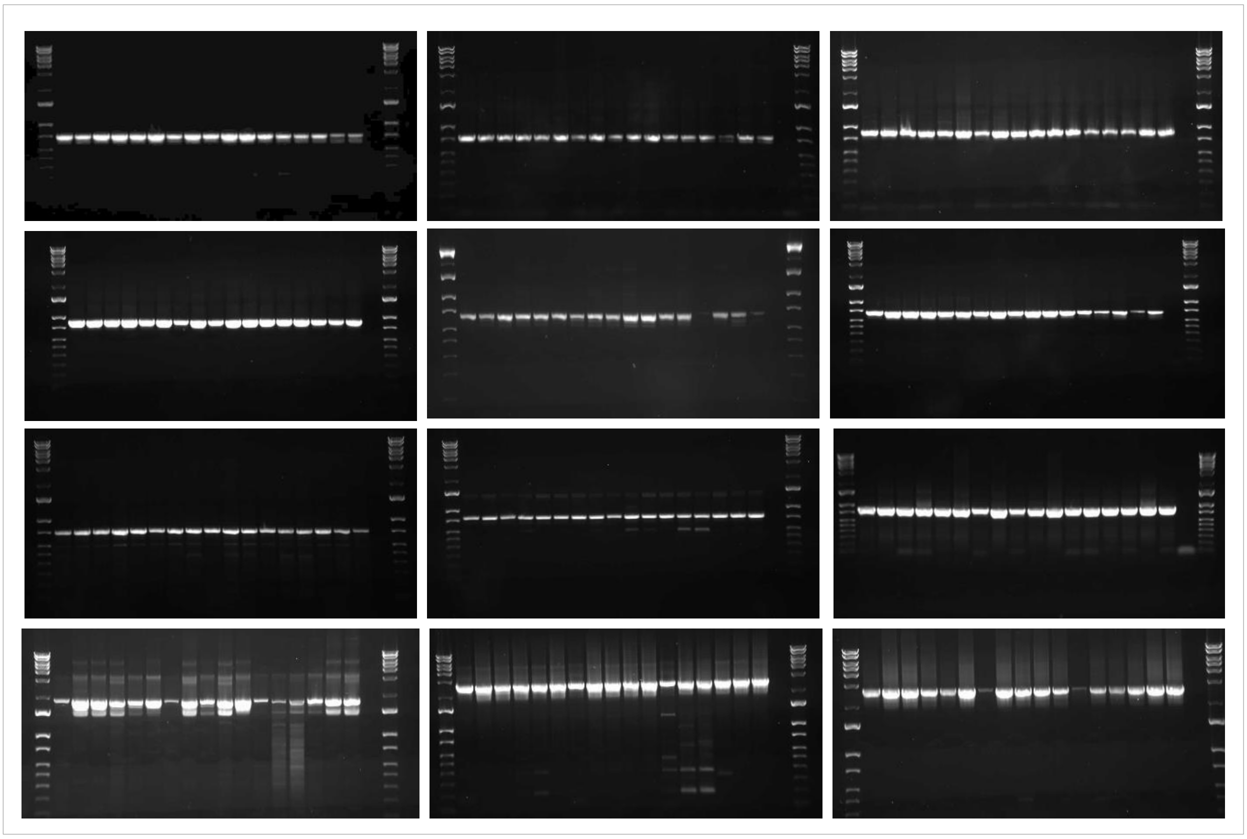

Figure S5. PCR amplification of 12 NUMT regions with genomic DNA from Dominette and other 7 breeds in a total of 17 animals[1].

One NUMT region is presented in each gel, from left to right:

- on the first row: UMD_14.1, UMD_2.5 and UMD_10.8;
- on the second row: UMD_15.B, UMD_28.2 and UMD_1.5;
- on the third row: UMD_27.1, UMD_8.6 and UMD_15.7;
- on the forth row UMD_11.3, UMD_18.9 and UMD_4.5.

ExactLadder DNA PreMix 2 Log DNA marker (Ozyme) was used in all gels (lanes 1 and 20). On lane 19, the negative control was charged.

1 Breeds: Hereford (1), Montbéliarde (3), Holstein (3), Normande (2), Limousine (1), Salers (2), Blonde d'Aquitaine (3), and Aubrac (2). Number of samples between brackets. Breeds are listed in the order of the amplicons on the gel.

Figure S6. Alignment of sequences obtained for amplicon UMD_15.B.

```
                          .    :    .    :    .    :    .    :    .    :    .    :
MON_UMD_15B-R+        AAAAAAAGTTTTAATTCTCTCCCTTTCCCTTATTACACTAATGTTAAACTTATGTCTACT
NOR_UMD_15B-R+                                                     AAACTTATGTCTACT
AUB_UMD_15B-R+                                                     AAACTTATGTCTACT
                      ____________________________________________________________
consensus             AAAAAAAGTTTTAATTCTCTCCCTTTCCCTTATTACACTAATGTTAAACTTATGTCTACT

                          .    :    .    :    .    :    .    :    .    :    .    :
MON_UMD_15B-R+        TAATTTCCATGAGTAATCTCCATAACAACAAAATTACTAAAAAATAAAGATCAACCACCA
NOR_UMD_15B-R+        TAATTTCCATGAGTAATCTCCATAACAACAAAATTACTAAAAAATAAAGATCAACCACCA
NOR_UMD_15B-F-                                       AATTACTAAAAAATAAAGATCAACCACCA
AUB_UMD_15B-R+        TAATTTCCATGAGTAATCTCCATAACAACAAAATTACTAAAAAATAAAGATCAACCACCA
AUB_UMD_15B-F-                                   ACAAAATTACTAAAAAATAAAGATCAACCACCA
BLA_UMD_15B-F-                                             TAAAAAATAAAGATCAACCACCA
HOL_UMD_15B-R+            TTCCATGAGTAATCTCCATAACAACAAAATTACTAAAAAATAAAGATCAACCACCA
                      ____________________________________________________________
consensus             TAATTTCCATGAGTAATCTCCATAACAACAAAATTACTAAAAAATAAAGATCAACCACCA

                          .    :    .    :    .    :    .    :    .    :    .    :
MON_UMD_15B-R+        ACCACCACTCATCATAGCTATATCTGCAGCTATTCCTATAGTATCTTCACTAAAAATCCA
NOR_UMD_15B-R+        ACCACCACTCATCATAGCTATATCTGCAGCTATTCCTATAGTATCTTCACTAAAAATCCA
NOR_UMD_15B-F-        ACCACCACTCATCATAGCTATATCTGCAGCTATTCCTATAGTATCTTCACTAAAAATCCA
AUB_UMD_15B-R+        ACCACCACTCATCATAGCTATATCTGCAGCTATTCCTATAGTATCTTCACTAAAAATCCA
AUB_UMD_15B-F-        ACCACCACTCATCATAGCTATATCTGCAGCTATTCCTATAGTATCTTCACTAAAAATCCA
BLA_UMD_15B-F-        ACCACCACTCATCATAGCTATATCTGCAGCTATTCCTATAGTATCTTCACTAAAAATCCA
HOL_UMD_15B-R+        ACCACCACTCATCATAGCTATATCTGCAGCTATTCCTATAGTATCTTCACTAAAAATCCA
                      ____________________________________________________________
consensus             ACCACCACTCATCATAGCTATATCTGCAGCTATTCCTATAGTATCTTCACTAAAAATCCA

                          .    :    .    :    .    :    .    :    .    :    .    :
MON_UMD_15B-R+        GCATCTCCAGTATCATAAATTACTCAGTCTCCTATGCCATAAAAGTCAAATACCAATGCC
NOR_UMD_15B-R+        GCATCTCCAGTATCATAAATTACTCAGTCTCCTATGCCATAAAAGTCAAATACCAATGCC
NOR_UMD_15B-F-        GCATCTCCAGTATCATAAATTACTCAGTCTCCTATGCCATAAAAGTCAAATACCAATGCC
AUB_UMD_15B-R+        GCATCTCCAGTATCATAAATTACTCAGTCTCCTATGCCATAAAAGTCAAATACCAATGCC
AUB_UMD_15B-F-        GCATCTCCAGTATCATAAATTACTCAGTCTCCTATGCCATAAAAGTCAAATACCAATGCC
BLA_UMD_15B-F-        GCATCTCCAGTATCATAAATTACTCAGTCTCCTATGCCATAAAAGTCAAATACCAATGCC
HOL_UMD_15B-R+        GCATCTCCAGTATCATAAATTACTCAGTCTCCTATGCCATAAAAGTCAAATACCAATGCC
                      ____________________________________________________________
consensus             GCATCTCCAGTATCATAAATTACTCAGTCTCCTATGCCATAAAAGTCAAATACCAATGCC

                          .    :    .    :    .    :    .    :    .    :    .    :
MON_UMD_15B-R+        ATCTCATTGTTTTTATAAATCACAGAACTACTGAAACTTCTATTAATAAGCCCAAAAGAA
NOR_UMD_15B-R+        ATCTCATTGTTTTTATAAATCACAGAACTACTGAAACTTCTATTAATAAGCCCAAAAGAA
NOR_UMD_15B-F-        ATCTCATTGTTTTTATAAATCACAGAACTACTGAAACTTCTATTAATAAGCCCAAAAGAA
AUB_UMD_15B-R+        ATCTCATTGTTTTTATAAATCACAGAACTACTGAAACTTCTATTAATAAGCCCTAAAGAA
AUB_UMD_15B-F-        ATCTCATTGTTTTTATAAATCACAGAACTACTGAAACTTCTATTAATAAGCCCTAAAGAA
BLA_UMD_15B-F-        ATCTCATTGTTTTTATAAATCACAGAACTACTGAAACTTCTATTAATAAGCCCTAAAGAA
HOL_UMD_15B-R+        ATCTCATTGTTTTTATAAATCACAGAACTACTGAAACTTCTATTAATAAGCCCAAAAGAA
BLA_UMD_15B-R+                          ATCACAGAACTACTGAAACTTCTATTAATAAGCCCTAAAGAA
                      ____________________________________________________________
consensus             ATCTCATTGTTTTTATAAATCACAGAACTACTGAAACTTCTATTAATAAGCCCAAAAGAA

                          .    :    .    :    .    :    .    :    .    :    .    :
MON_UMD_15B-R+        ACATTCCTAAAACGACAATAATGAAACTCAAATATCAGGGTATTCTTTTAGTAGCTATAG
NOR_UMD_15B-R+        ACATTCCTAAAACGACAATAATGAAACTCAAATATCAGGGTATTCTTTTAGTAGCTATAG
NOR_UMD_15B-F-        ACATTCCTAAAACGACAATAATGAAACTCAAATATCAGGGTATTCTTTTAGTAGCTATAG
AUB_UMD_15B-R+        ACATTCCTAAAACGACAATAATGAAACTCAAATATCAGGGTATTCTTTTAGTAGCTATAG
AUB_UMD_15B-F-        ACATTCCTAAAACGACAATAATGAAACTCAAATATCAGGGTATTCTTTTAGTAGCTATAG
BLA_UMD_15B-F-        ACATTCCTAAAACGACAATAATGAAACTCAAATATCAGGGTATTCTTTTAGTAGCTATAG
HOL_UMD_15B-R+        ACATTCCTAAAACGACAATAATGAAACTCAAATATCAGGGTATTCTTTTAGTAGCTATAG
BLA_UMD_15B-R+        ACATTCCTAAAACGACAATAATGAAACTCAAATATCAGGGTATTCTTTTAGTAGCTATAG
                      ____________________________________________________________
consensus             ACATTCCTAAAACGACAATAATGAAACTCAAATATCAGGGTATTCTTTTAGTAGCTATAG

                          .    :    .    :    .    :    .    :    .    :    .    :
MON_UMD_15B-R+        CTGTGGTATAACAAGAAGTCACCAATATTCCCTCTCTTTCTGCACTTTTTGACAACTGAC
NOR_UMD_15B-R+        CTGTGGTATAACAAGAAGTCACCAATATTCCCTCTCTTTCTGCACTTTTTGACAACTGAC
NOR_UMD_15B-F-        CTGTGGTATAACAAGAAGTCACCAATATTCCCTCTCTTTCTGCACTTTTTGACAACTGAC
```

```
AUB_UMD_15B-R+        CTGTGGTATAACAAGAAGTCACCAATATTCCCTCTCTTTCTGCACTTTTTGACAACTGAC
AUB_UMD_15B-F-        CTGTGGTATAACAAGAAGTCACCAATATTCCCTCTCTTTCTGCACTTTTTGACAACTGAC
BLA_UMD_15B-F-        CTGTGGTATAACAAGAAGTCACCAATATTCCCTCTCTTTCTGCACTTTTTGACAACTGAC
HOL_UMD_15B-R+        CTGTGGTATAACAAGAAGTCACCAATATTCCCTCTCTTTCTGCACTTTTTGACAACTGAC
BLA_UMD_15B-R+        CTGTGGTATAACAAGAAGTCACCAATATTCCCTCTCTTTCTGCACTTTTTGACAACTGAC
                      ____________________________________________________________
consensus             CTGTGGTATAACAAGAAGTCACCAATATTCCCTCTCTTTCTGCACTTTTTGACAACTGAC

                          .    :    .    :    .    :    .    :    .    :    .    :
MON_UMD_15B-R+        AGTTGAAAAGAGGCCTTGAGAAACAGGGGTTTAGGAAGAATACTAAGTTTAGAACTCTCC
NOR_UMD_15B-R+        AGTTGAAAAGAGGCCTTGAGAAACAGGGGTTTAGGAAGAATACTAAGTTTAGAACTCTCC
NOR_UMD_15B-F-        AGTTGAAAAGAGGCCTTGAGAAACAGGGGTTTAGGAAGAATACTAAGTTTAGAACTCTCC
AUB_UMD_15B-R+        AGTTGAAAAGAGGCCTTGAGAAACAGGGGTTTAGGAAGAATACTAAGTTTAGAACTCTCC
AUB_UMD_15B-F-        AGTTGAAAAGAGGCCTTGAGAAACAGGGGTTTAGGAAGAATACTAAGTTTAGAACTCTCC
BLA_UMD_15B-F-        AGTTGAAAAGAGGCCTTGAGAAACAGGGGTTTAGGAAGAATACTAAGTTTAGAACTCTCC
HOL_UMD_15B-R+        AGTTGAAAAGAGGCCTTGAGAAACAGGGGTTTAGGAAGAATACTAAGTTTAGAACTCTCC
BLA_UMD_15B-R+        AGTTGAAAAGAGGCCTTGAGAAACAGGGGTTTAGGAAGAATACTAAGTTTAGAACTCTCC
                      ____________________________________________________________
consensus             AGTTGAAAAGAGGCCTTGAGAAACAGGGGTTTAGGAAGAATACTAAGTTTAGAACTCTCC

                          .    :    .    :    .    :    .    :    .    :    .    :
MON_UMD_15B-R+        ACCCATCTACCTATGCACCTGATTATTCATCTGTGAATCCATCCATCCATGCACAACCTA
NOR_UMD_15B-R+        ACCCATCTACCTATGCACCTGATTATTCATCTGTGAATCCATCCATCCATGCACAACCTA
NOR_UMD_15B-F-        ACCCATCTACCTATGCACCTGATTATTCATCTGTGAATCCATCCATCCATGCACAACCTA
AUB_UMD_15B-R+        ACCCATCTACCTATGCACCTGATTATTCATCTGTGAATCCATCCATCCATGCACAACCTA
AUB_UMD_15B-F-        ACCCATCTACCTATGCACCTGATTATTCATCTGTGAATCCATCCATCCATGCACAACCTA
BLA_UMD_15B-F-        ACCCATCTACCTATGCACCTGATTA
HOL_UMD_15B-R+        ACCCATCTACCTATGCACCTGATTATTCATCTGTGAATCCATCCATCCATGCACAACCTA
BLA_UMD_15B-R+        ACCCATCTACCTATGCACCTGATTATTCATCTGTGAATCCATCCATCCATGCACAACCTA
                      ____________________________________________________________
consensus             ACCCATCTACCTATGCACCTGATTATTCATCTGTGAATCCATCCATCCATGCACAACCTA

                          .    :    .    :    .    :    .    :    .    :    .    :
MON_UMD_15B-R+        TCTGTCCATCTTTCCATCTACCCAACCCTCTTTAACAAATATTTATTGAGAATATACTAT
NOR_UMD_15B-R+        TCTGTCCATCTTTCCATCTACCCAACCCTCTTTAACAAATATTTATTGAGAATATACTAT
NOR_UMD_15B-F-        TCTGTCCATCTTTCCATCTACCCAACCCTCTTTAACAAATATTTATTGAGAATATACTAT
AUB_UMD_15B-R+        TCTGTCCATCTTTCCATCTACCCAACCCTCTTTAACAAATATTTATTGAGAATATACTAT
AUB_UMD_15B-F-        TCTGTCCATCTTTCCATCTACCCAACCCTCTTTAACAAATATTTATTGAGAATATACTAT
HOL_UMD_15B-R+        TCTGTCCATCTTTCCATCTACCCAACCCTCTTTAACAAATATTTATTGAGAATATACTAT
BLA_UMD_15B-R+        TCTGTCCATCTTTCCATCTACCCAACCCTCTTTAACAAATATTTATTGAGAATATACTAT
                      ____________________________________________________________
consensus             TCTGTCCATCTTTCCATCTACCCAACCCTCTTTAACAAATATTTATTGAGAATATACTAT

                          .    :    .    :    .    :    .    :    .    :    .    :
MON_UMD_15B-R+        GGACTCCCAGTAGGGATACAGAAGTGAATAAAGTAGAAATCAGAGAAATCTGGGCTGATG
NOR_UMD_15B-R+        GGACTCCCAGTAGGGATACAGAAGTGAATAAAGTAGAAATCAGAGAAATCTGGGCTGATG
NOR_UMD_15B-F-        GGACTCCCAGTAGGGATACAGAAGTGAATAAAGTAGAAATCAGAGAAATCTGGGCTGATG
AUB_UMD_15B-R+        GGACTCCCAGTAGGGATACAGAAGTGAATAAAGTAGAAATCAGAGAAATCTGGGCTGATG
AUB_UMD_15B-F-        GGACTCCCAGTAGGGATACAGAAGTGAATAAAGTAGAAATCAGAGAAATCTGGGCTGATG
HOL_UMD_15B-R+        GGACTCCCAGTAGGGATACAGAAGTGAATAAAGTAGAAATCAGAGAAATCTGGGCTGATG
BLA_UMD_15B-R+        GGACTCCCAGTAGGGATACAGAAGTGAATAAAGTAGAAATCAGAGAAATCTGGGCTGATG
                      ____________________________________________________________
consensus             GGACTCCCAGTAGGGATACAGAAGTGAATAAAGTAGAAATCAGAGAAATCTGGGCTGATG

                          .    :    .    :    .    :    .    :    .    :    .    :
MON_UMD_15B-R+        TCATAACTCAGTCACTTAATACAACTGTGTTACCTTGTAAAGTTTTGTGTTCTCTTGAGC
NOR_UMD_15B-R+        TCATAACTCAGTCACTTAATACAACTGTGTTACCTTGTAAAGTTTTGTGTTCTCTTGAGC
NOR_UMD_15B-F-        TCATAACTCAGTCACTTAATACAACTGTGTTACCTTGTAAAGTTTTGTGTTCTCTTGAGC
AUB_UMD_15B-R+        TCATAACTCAGTCACTTAATACAACTGTGTTACCTTGTAAAGTTTTGTGTTCTCTTGAGC
AUB_UMD_15B-F-        TCATAACTCAGTCACTTAATACAACTGTGTTACCTTGTAAAGTTTTGTGTTCTCTTGAGC
HOL_UMD_15B-R+        TCATAACTCAGTCACTTAATACAACTGTGTTACCTTGTAAAGTTTTGTGTTCTCTTGAGC
BLA_UMD_15B-R+        TCATAACTCAGTCACTTAATACAACTGTGTTACCTTGTAAAGTTTTGTGTTCTCTTGAGC
                      ____________________________________________________________
consensus             TCATAACTCAGTCACTTAATACAACTGTGTTACCTTGTAAAGTTTTGTGTTCTCTTGAGC

                          .    :    .    :    .    :    .    :    .    :    .    :
MON_UMD_15B-R+        TTCTGTCTTCATCTGTAAATTTTGGATTCTAACTCTGCTTCACAGTGAAAAGGATTAGAA
NOR_UMD_15B-R+        TTCTGTCTTCATCTGTAAATTTTGGATTCTAACTCTGCTTCACAGTGAAAAGGATTAGAA
NOR_UMD_15B-F-        TTCTGTCTTCATCTGTAAATTTTGGATTCTAACTCTGCTTCACAGTGAAAAGGATTAGAA
AUB_UMD_15B-R+        TTCTGTCTTCATCTGTAAATTTTGGATTCTAACTCTGCTTCACAGTGAAAAGGATTAGAA
AUB_UMD_15B-F-        TTCTGTCTTCATCTGTAAATTTTGGATTCTAACTCTGCTTCACAGTGAAAAGGATTAGAA
```

```
HOL_UMD_15B-R+         TTCTGTCTTCATCTGTAAATTTTGGATTCTAACTCTGCTTCACAGTGAAAAGGATTAGAA
BLA_UMD_15B-R+         TTCTGTCTTCATCTGTAAATTTTGGATTCTAACTCTGCTTCACAGTGAAAAGGATTAGAA
                       ____________________________________________________________
consensus              TTCTGTCTTCATCTGTAAATTTTGGATTCTAACTCTGCTTCACAGTGAAAAGGATTAGAA

                           .    :    .    :    .    :    .    :    .    :    .    :
MON_UMD_15B-R+         GAAATACTGGACTTATATAGAAAACAGCCCAGTGGCAAGAGCATTGTAGGTACCCCC
NOR_UMD_15B-R+         GAAATACTGGACTTATATAGAAAACAGCCCAGTGGCAAGAGCATTGTAGGTACCCCC
NOR_UMD_15B-F-         GAAATACTGGACTTATATAGA
AUB_UMD_15B-R+         GAAATACTGGACTTATATAGAAAACAGCCCAGTGGCAAGAGCATTGTAGGTACCCCC
AUB_UMD_15B-F-         GAAATACTGGACTTAT
HOL_UMD_15B-R+         GAAATACTGGACTTATATAGAAAACAGCCCAGTGGCAAGAGCATTGTAGGTACCCCC
BLA_UMD_15B-R+         GAAATACTGGACTTATATAGAAAACAGCCCAGTGGCAAGAGCATTGTAGGTACCCCCCAA
                       ____________________________________________________________
consensus              GAAATACTGGACTTATATAGAAAACAGCCCAGTGGCAAGAGCATTGTAGGTACCCCCCAA

                           .    :    .    :    .    :    .    :    .    :    .    :
BLA_UMD_15B-R+         ACAGTAGCTTTGCATGTGGG
                       ____________________________________________________________
consensus              ACAGTAGCTTTGCATGTGGG
```
